# ATP5O Hypo-crotonylation Caused by HDAC2 Hyper-Phosphorylation Is a Primary Detrimental Factor for Downregulated Phospholipid Metabolism under Chronic Stress

**DOI:** 10.34133/2022/9834963

**Published:** 2022-11-24

**Authors:** Liang-Jian Chen, Zhi-Yuan Tu, Yang Wang, Yu-Hao He, Xin Wang, Shu-Zhen Tao, Yang-Yang Xu, Cong-Rong Li, Ruo-Lei Wang, Zhi-Xia Yang, Jing Sun, Xiang Ma, Dong Zhang

**Affiliations:** ^1^ State Key Lab of Reproductive Medicine, Nanjing Medical University, Nanjing, 211166 Jiangsu, China; ^2^ Department of Obstetrics and Gynecology, Reproductive Medicine Center, The First Affiliated Hospital of Anhui Medical University, Hefei 230022, China; ^3^ State Key Laboratory of Reproductive Medicine, the Center for Clinical Reproductive Medicine, The First Affiliated Hospital of Nanjing Medical University, Nanjing, 210029, China; ^4^ Department of Psychiatry, Nanjing Brain Hospital affiliated to Nanjing Medical University, Nanjing, 210029 Jiangsu, China; ^5^ Animal Core Facility, Nanjing Medical University, Nanjing, 211166, Jiangsu, P .R., China

## Abstract

*Objective.* Chronic stress (CS)-induced abnormal metabolism and other subsequent aspects of abnormality are threatening human health. Little is known regarding whether and how protein post-translational-modifications (PTMs) correlate with abnormal metabolism under CS. The aim of this study was to address this issue and also identify novel key protein PTM. *Methods.* First, we screened which pan-PTM had significant change between control and CS female mice and whether clinical CS females had similar pan-PTM change. Second, we performed quantitative PTM-omics and metabolomics to verify the correlation between abnormal protein PTMs and atypical metabolism. Third, we performed quantitative phospho-omics to identify the key PTM-regulating enzyme and investigate the interaction between PTM protein and PTM-regulating enzyme. Fourth, we attempted to rectify the abnormal metabolism by correcting the activity of the PTM-regulating enzyme. Finally, we examined whether the selected key protein was also correlated with stress scores and atypical metabolism in clinical women. *Results.* We initially found that multiple tissues of CS female mice have downregulated pan-crotonylation, and verified that the plasma of clinical CS females also had downregulated pan-crotonylation. Then we determined that ATP5O-K51 crotonylation decreased the most and also caused gross ATP5O decrement, whereas the plasma of CS mice had downregulated phospholipids. Next, downregulating ATP5O crotonylation partially recapitulated the downregulated phospholipid metabolism in CS mice. Next, we verified that HDAC2-S424 phosphorylation determined its decrotonylation activity on ATP5O-K51. Furthermore, correcting HDAC2 hyper-phosphorylation recovered the gross ATP5O level and partially rescued the downregulated phospholipid metabolism in CS mice. Finally, the ATP5O level was also significantly lower and correlated with high stress scores and downregulated phospholipid metabolism in clinical female plasma. *Conclusion.* This study discovered a novel PTM mechanism involving two distinct types of PTM in CS and provided a novel reference for the clinical precautions and treatments of CS.

## 1. Introduction

Stress, including acute and chronic forms, impacts approximately 275 million people worldwide, or 4% of the population, among which 62% are women [ 1– 3]. Although the immediate effects of chronic stress (CS) are small, they can accumulate, and persistent negative CS can cause significant damage to various aspects of body functions, including cardiovascular [ 4], neuron [ 5], immune [ 6], endocrine [ 7], gut microbiota [ 8], and metabolism. Among these effects, abnormal metabolism appears to be a more fundamental problem that subsequently impairs the normal function of other aspects.

Lipids are the main components of the cell membrane or organelle membrane, the essential substances for many important biochemical reactions, and important energy storage and providers. Lipids include glyceride, phospholipid, sphingolipid, and cholesterol fat. The abnormal metabolism of these lipids significantly impacts diverse aspects of physical functions. For example, for glyceride, being underweight or morbidly obese in NSCLC (non-small-cell lung cancer) and SCLC (small cell lung cancer) patients is associated with lower stage-specific survival [ 9]. Obesity remains a risk factor for cardiovascular disease even for metabolically healthy women [ 10]. Regarding phospholipids, ApoE4-induced phospholipid dysregulation is a critical factor involved in the AD (Alzheimer’s disease)-related cognitive deficits [ 11]. Phospholipids are essential for activating PPAR *α* (peroxisome proliferator-activated receptor *α*), which regulates lipid metabolism; deletion of CEPT1 (choline-ethanolamine phosphotransferase 1), which mediates phospholipogenesis, reduced perfusion and angiogenesis in ischemic hind limbs of a diabetic mouse model [ 12]. Regarding sphingolipids, the accumulation of sphingolipids increased mitochondrial reactive oxygen species (ROS) and reduced mitochondrial mass [ 13]. Decreased de novo sphingolipid synthesis increased bronchial reactivity in the absence of inflammation in the lung on the airway [ 14]. For cholesterol fat, the upregulation of brain cholesterol levels led to impaired mitophagy, a major pathology of diverse neurodegenerative diseases [ 15]. Cholesterol decrement through oral atorvastatin or dietary restriction inhibited monocyte infiltration and reversed macrophage accumulation in atherosclerotic plaques [ 16].

Notably, it has been shown that lipid metabolism was dysregulated under CS. For example, the hippocampus lipidome of CS rats showed a decrease in PE (phosphatidylethanolamine), PC (phosphatidylcholine), SM (sphingomyelin), and dhSM (dihydrosphingomyelin) and an increase in detrimental LPE (lysophosphatidylethanolamine) and LPC (lysophosphatidylcholine) [ 17]. Liquid high-fructose supplementation increased IHTG (intrahepatic-triglycerides) and VLDL-TG (very-low-density lipoprotein-triglycerides) secretion after the feeding phase, whereas chronic stress potentiates the effects of high fructose on the export of newly synthesized VLDL-TGs [ 18]. Many studies have investigated the transcriptional [ 19, 20], translational [ 21], and epigenetic [ 22, 23] regulation of lipid metabolism under CS.

Posttranslational modification (PTM) is the most extensive way to activate/inactivate proteins, which ultimately execute various cellular functions. Several studies have shown that CS causes dysregulation of several types of histone PTMs [ 24, 25], but until now, nothing has been known about the correlation between PTMs and lipid metabolism. In this study, using female mice under CS, we found that ATP5O hypo-crotonylation is a critical factor for abnormal phospholipid metabolism and is regulated by HDAC2 hyper-phosphorylation. Moreover, the ATP5O level was highly correlated with the abnormal phospholipid metabolism of clinical CS females.

## 2. Material and Methods

### 2.1. Animal Models

Animal experimental procedures in our study were all approved by Institutional Animal Care and Use Committee (IACUC) of Nanjing Medical University (NJMU), the approval No. is IACUC-2005003. All mice were housed under standard specific pathogen-free (SPF) conditions of ACF (Animal Core Facility). For the acquisition of ovaries or other tissues, mice were first anesthetized with CO _2_, and then sacrificed by cervical dislocation.

For chronic stress (CS) model female mice, a stainless steel cylinder (15 cm in length and 2.5 cm in diameter, Figure 2(a)) was used (one cylinder for one mouse). The female mouse was hold in the cylinder for six hours per day, then released into standard cage. The treatment started from 9 : 00 am every day.

For all fertility assays, 10 control and 10 CS ICR female mice were used. WT mating male mice were monthly rotated between cages according to a random allocation table (Supp. table 1), mating started with 2-month-old control or CS female mice and control male mice (all are ICR stains).

### 2.2. Human Subject Questionnaire and Assays

All-human-related questionnaire and assays were approved by the Human Medical Ethics Committee (HMEC) of Jiangsu Provincial Hospital affiliated to Nanjing Medical University, the approval No. is 2019-SR-227.

To score the stress level of female subjects, a Hamilton Anxiety Rating Scale (HARC) was used, and each subject was apprised by two independent psychologists and the final score was the average score of two psychologists.

The sera of the subjects were used for quantitative lipidome and the assays of biochemical indexes.

### 2.3. TMT-Labeling Quantitative Phosphoproteomics and Label-Free Quantitative Crotonylomics

For phosphoproteomics, about sixty two-month-old ovaries per repeat (about 300 mg), two repeats for WT and CS, respectively, were sent to PTM Biolab LLC (Hangzhou, Zhejiang, China). In brief, ovaries were cracked and the supernatant was digested by trypsin into peptides. Then, the peptides from individual samples were isobaric-mass tagged by TMT ^6^-126, 127, 128, and 129, respectively, according to the manufacturer’s protocol for TMT kit (Thermo Fisher). Next, TMT-labeled tryptic peptides were fractionated by high pH reverse-phase HPLC using a Thermo Betasil C18 column, then peptide fractions were subjected to NSI source followed by tandem mass spectrometry (MS/MS) in Q ExactiveTM Plus (Thermo) coupled online to the UPLC for identification of peptides (for proteomics). The resulting MS/MS data were processed using the Maxquant search engine (v.1.5.2.8) for peptides or phosphorylation sites (for phosphoproteomics). Peptides were enriched by IMAC microspheres.

For crotonylomics, sample preparation, processing, peptide identification were all the same as above except that the samples were not isobaric-mass tagged, and the peptides were enriched by crotonylated resin (Cat No. PTM503).

Secondary mass spectrometry data were searched using Maxquant (v1.6.6.0). The searching parameter settings were as follows: the database was mouse SwissPort (17032 sequences), antidatabase was added to calculate the false positive rate (FDR) caused by random matching, and a common pollution library was added to the database to eliminate the effect of contaminating proteins in the identification results. The digestion mode was set to Trypsin/P; the number of missed digested sites was set to 4; the minimum length of the peptide was set to 7 amino acid residues; the maximum number of peptide modifications was set to 5; and the mass error tolerance of the primary precursor ion of the first search and main search was set to 20 ppm and 20 ppm, respectively, and the mass error tolerance of the secondary fragment ion was 0.02 Da. Cysteine alkylation was set to fixed modification, variable modification to oxidation of methionine, acetylation of the N-terminus of the protein, and crotonylation of lysine. The FDR for protein identification and PSM identification was set to 1%.

For quantification, the raw LC-MS datasets were first searched against database and converted into matrices containing reporter intensity of peptides across samples. The relative quantitative value of each modified peptide was then calculated based on the intensity information by the following steps:

First, the intensities of modified peptides ( I ) were centralized and transformed into relative quantitative values ( U ) of modified peptides in each sample. The formula is listed as follows: i denotes the sample and j denotes the modified peptide.

(1)Rij=IijMeanIj.

Next, as both Proteomics and Posttranslational modification profiling were conducted on the same cohort, the relative quantitative value of the modified peptide is usually divided by the relative quantitative value of corresponding protein to remove the influence from protein expression of modifications.

For significant difference selection, the fold change (FC) was calculated as the mean of the ratio of each modification site within the repeats. For example, to calculate the fold change between sample A and sample B, the formula is listed as following: R denotes the relative quantitative value of the modification site, i denotes the sample and k denotes the modification site.

(2)FCA/B,k=MeanRik,i∈AMeanRik,i∈B.

To calculate the statistical significance of the difference between groups, the coefficient of variation (CV) for each site in the two comparison groups was calculated as a significance index. The formula is listed as following:

(3)CV=SDA1k/B1k,A2k/B2kMeanA1k/B1k,A2k/B2k.

The modification site with CV<0.1 and fold change > 1.3 (crotonylomics, supplementary dataset 1) or 1.2 (phosphoproteomics, supplementary dataset 4) was regarded as significant upregulated site, while the modification site with CV < 0.1 and fold change < 0.77 (crotonylomics, supplementary dataset 1) or < 0.833 (phosphoproteomics, supplementary dataset 4) was regarded as significant down-regulated site. The reason we set a smaller fold change as the difference threshold for phosphoproteomics is that TMT labeling tends to decrease actual fold change.

### 2.4. Label-Free Quantitative Metabolomics

Metabonomics were all done by Wuhan Metware Biotechnology Co. (Wuhan, Hubei, China). Five to twelve repeats were done on the metabonomics of human female serum or female mouse serum. Full spectrum metabolome, which cover all kinds of metabolites, were used for the initial screen of significantly different metabolites between control and CS female mice. Then we found that lipidome had the largest percentage of discrepancy, thereby lipidome was done for the difference between control vs. ATP5O crotonylation inhibition in mice, CS vs. HDAC2 phosphorylation inhibition in mice, and control females vs. females with medium stress score vs. females with high stress score in human.

The sample extracts were analyzed using an LC-ESI-MS/MS system (UPLC, ExionLC AD’ https://sciex.com.cn/; MS, QTRAP® System, https://sciex.com/). The Triple TOF mass spectrometer was used for its ability to acquire MS/MS spectra on an information dependent basis (IDA) during an LC/MS experiment. In this mode, the acquisition software (TripleTOF 6600, AB SCIEX) continuously evaluates the full scan survey MS data as it collects and triggers the acquisition of MS/MS spectra depending on preselected criteria. In each cycle, 12 precursor ions whose intensity greater than 100 were chosen for fragmentation at collision energy (CE) of 30 V (12 MS/MS events with product ion accumulation time of 50 msec each). ESI source conditions were set as following: ion source gas 1 as 50 Psi, ion source gas 2 as 50 PSI, as 25 PSI, source temperature 500°C, Ion Spray Voltage Floating (ISVF) 5500 V or -4500 V in positive or negative modes, respectively.

Next, based on the company’s self-built standard target metabolite database, MWDB (Metware database), the information and secondary spectrum data are qualitatively analyzed according to the retention time RT (retention time) and daughter/parent ions of the detected substance.

Then, the metabolites were quantified by multiple reaction monitoring (MRM) of the triple quadrupole mass spectrometry machine. In detail, In MRM mode, the first quadrupole screens the precursor ions (parent ions) of the target substance, and excludes the precursor ions corresponding to other substances to preliminarily eliminate interference; secondly, the precursor ions are broken up after collision-induced ionization in the second quadrupole, and form a series of fragment ions unique to the substance according to the structural characteristics of the substance itself; thirdly, the fragment ions are filtered through the third quadrupole, a typical characteristic fragment ion was selected and the nontarget ion was excluded, which ensured more accurate quantification and better repeatability. Finally, the output data from different samples were obtained, the area under the chromatographic peaks of all extracted ions of a metabolite was integrated as the quantitative data of the metabolite.

Significantly regulated metabolites between groups were determined by VIP (variable importance in projection) ≥1 and absolute Log2FC (fold change) ≥1 . VIP values were extracted from OPLS-DA (Orthogonal Partial Least Squares-Discriminant Analysis) result, which also contain score plots and permutation plots, was generated using R package MetaboAnalystR. The data was log transform (log2) and mean centering before OPLS-DA. In order to avoid overfitting, a permutation test (200 permutations) was performed.

### 2.5. Antibodies

Pan-PTM antibodies: Mouse anti-Crotonyllysine monoclonal antibody (Cat#: PTM-502; PTM BioLab, Hangzhou, China); Mouse anti-Acetyllysine monoclonal antibody (Cat#: PTM-101; PTM BioLab); Rabbit anti-Malonyllysine polyclonal antibody (Cat#: PTM-901; PTM BioLab); Mouse anti-Benzoyllysine monoclonal antibody (Cat#: PTM-762; PTM BioLab); Mouse anti-Hydroxybutyryllysine monoclonal antibody (Cat#: PTM-802; PTM BioLab); Rabbit anti-Lactyllysine polyclonal antibody (Cat#: PTM-1401; PTM BioLab); and Rabbit anti-Succinyllysine polyclonal antibody (Cat#: PTM-401; PTM BioLab). Each of these pan-PTM antibodies was made against lysine with a certain type of PTM modification above. Presumably they can detect most of modified lysine and have been widely used to detect pan-PTMs within diverse tissues [ 26– 29].

Other primary antibodies: Mouse monoclonal anti-GAPDH (Cat#: 30201ES60; YEASEN, Shanghai, China); mouse monoclonal anti- *β*-Actin (Cat#: A5316-100; Sigma, MS, USA); Mouse monoclonal anti- *β*-Tubulin (Cat#: sc-5274; Santa Cruz, TX, USA); Mouse monoclonal anti-alpha Tubulin (Acetyl Lys40) (cat#: bsm-33235 M; Bioss, Beijing, China); Rabbit anti-Transferrin polyclonal antibody (Cat#: 17435-1-ap; Proteintech, Chicago, USA); Rabbit anti-ATP5O polyclonal antibody (Cat#: D126152; BBI Life science, Shanghai, China); Rabbit anti-ATP5A1 polyclonal antibody (Cat#: D154243; BBI Life science, Shanghai, China); Rabbit anti-ATPB polyclonal antibody (Cat#: A5286; Selleckchem, Shanghai, China); Mouse anti-COX4L1 monoclonal antibody (Cat#: D190618; BBI Life science, Shanghai, China); Anti-AKT (Ab-129) Rabbit polyclonal antibody (Cat#: D151616-0100; BBI Life science, Shanghai, China); Rabbit anti-Phosphpho AKT (Ser473, Cat# 4060, Cell Signaling Technology); Rabbit anti-Phosphpho RPS6 (Ser235/236, Cat#: D155178; BBI Life science, Shanghai, China); Rabbit anti-STAT5A polyclonal antibody (Cat#: D220085; BBI Life science, Shanghai, China); Rabbit anti-FAM126A polyclonal antibody (Cat#: bs-11554R; Bioss, Beijing, China); Rabbit anti-PTDSS1 polyclonal antibody (Cat#: BS-19583R; Bioss, Beijing, China); Rabbit anti-HDAC2 polyclonal antibody (Cat#: 12922-3-ap, Proteintech, Chicago, USA); Mouse monoclonal anti-strep II Tag (Cat#: YFMA0054, Yifeixue, Nanjing, China); Mouse monoclonal anti-flag Tag (Cat#: D190828, BBI Life science, Shanghai, China); Rabbit anti-HDAC1-S421 ^p^ polyclonal antibody (Cat#: AP59578, Abcepta, San Diego, CA, USA); Rabbit anti-Ubinuclein polyclonal antibody (Cat#: DF8873, Affinity Biosciences, Melbourne, Australia); Fibrinogen alpha chain (FGA) Rabbit pAb (Cat#: A1453, ABclonal, Wuhan, Hubei, China); Albumin Polyclonal antibody (Cat#: 16475-1-AP; Proteintech, Rosemont, IL, USA); AffiniPure Goat Anti-Mouse IgG (H + L) (Jackson, Cat#: 115-005-003, West Grove, PA, USA); Rabbit anti-ATP5O-K51 ^cr^ rabbit polyclonal antibody was made against ASK (crotonyl) EKKLDQVEKELLC by YuBo BioTech and purified by affinity purification; Rabbit anti-HDAC2-S424 ^p^,and anti-KAT7-S102 ^p^ polyclonal antibody was made against SDS(phospho)EDEGEGGRRC and CPLRQTRSSGS (phospho) ET by Zoonbio BioTech and purified by affinity purification.

Secondary antibodies: Horseradish peroxidase (HRP)-conjugated rabbit anti-goat IgG and HRP-conjugated goat anti-mouse IgG were purchased from Vazyme (Nanjing, Jiangsu, China). Cy2-conjugated donkey anti-rabbit IgG (Code: 711-225-152), Cy2-conjugated donkey anti-mouse IgG (Code: I715-225-150), and Rhodamine (TRITC)-conjugated donkey anti-rabbit IgG (Code: 711-025-152) were purchased from Jackson ImmunoResearch Laboratory (West Grove, PA, USA).

### 2.6. Competitive Peptides and Animal Injection

For all competitive peptides, the cell-penetrating peptide TAT sequence, CYGRKKRRQRRR, was fused to the protein-specific sequence. TAT peptide was used in all control groups.

For inhibition of endogenous ATP5O crotonylation at K51, the sequence is CYGRKKRRQRRRATALYSAASKEKKL and named as TAC (TAT-fused ATP5O crotonylation sequence).

For inhibition of endogenous HDAC2 phosphorylation at S424, the sequence is CYGRKKRRQRRRSDSEDEGEGGRR and named as THP (TAT-fused HDAC2 phosphorylation sequence).

For inhibition of endogenous KAT7 phosphorylation at S102, the sequence is CYGRKKRRQRRRSSGSETEQVVDF and named as TKP (TAT-fused KAT7 phosphorylation sequence).

For inhibition of endogenous CREBBP phosphorylation at S2361 & S2363, the sequence is CYGRKKRRQRRRQSQPPHSSPSPR and named as TCP (TAT-fused CREBBP phosphorylation sequence).

Each peptide was dissolved in sterile ultrapure water with 10% DMSO (Sigma) to a stock conc. of 5 mg/ml. For mouse injection, the stock peptide was diluted with 0.9% Nacl solution to a final conc. of 0.5 mg/ml and the dosage is 6 mg/kg. TAT-only sequence was used for control injection.

### 2.7. In Vivo Dose-Dependent ATP5O Decrotonylation by HDAC2

For assays in Figure 5(l)– 5(n), fixed amount (0.25 *μ*g) of ATP5O-WT-strepII plasmid (in pcDNA3.1) and increasing amount (0.25, 0.5, and 1 *μ*g) of HDAC2-WT-Flag plasmid were transfected into 293 T cells, and cells were cultured for 3 days for the expression of exogenous proteins. The cells were then lysed and subjected to western blot.

For assays in Figure 5(o)– 5(s), increasing amount (0.25, 0.5, 1 *μ*g) of ATP5O-WT-strepII plasmid and HDAC2-WT-Flag plasmid were transfected.

For assays in Figures 5(t) and 5(u), three different transfections (0.25 *μ*g ATP5O-WT-strepII plasmid alone, 0.25 *μ*g ATP5O-WT-strepII+0.25 *μ*g HDAC2-WT-Flag plasmid, and 0.25 *μ*g of both plasmids+MG132) were performed.

### 2.8. In Vivo ATP5O Ubiquitination Assay

For assays in Figures 5(v) and 5(w), we do not know yet the specific E3 ubiquitin ligase for ATP5O, therefore we used ATP5O antibody to do immunoprecipitation (as “Co-immunoprecipitaion” part in the “Chen LJ et al.-Second revision-supplementary information” file), eluted the immunocomplex (that contains ATP5O and its E3 ubiquitin ligase) with 0.2 M acidic glycine (pH 2.7), then buffer-exchanged the immunocomplex into 1 x ubiquitination reaction buffer and applied the immunocomplex into the ubiquitination reaction as reported before [ 30].

All other ubiquitination components were bought from R&D Systems Co. (Minneapolis, MN, USA). In vitro ubiquitination assays were set up in a 15 *μ*l ubiquitination reaction mixture including 10× reaction buffer, UBE1 (E1), UbcH5a/UBE2D1 (E2), CUL1/RBX1, SKP1/SKP2, Ubiquitin, ATP5O-E3 ubiquitin ligase complex, ATP, and MgSO _4_. The reaction mixture was incubated at 30°C for 2 hours. All information about the stock concentration (conc.), final conc., and final quantity are in Supp. table 5. Lastly, the reaction was disposed by western blot.

### 2.9. Animal/Individual Sample Inclusion, Experiment Grouping, Data Collection, and Data Analysis

Any selected oocyte has to be of normal quality (fully-grown oocyte from an antral follicle, normal diameter, tight connection between zona pellucida and oocyte membrane, etc.). Any selected female mouse has to be physically healthy (normal body weight, eats normally, normal activity, etc.). Any oocyte or mouse that is of bad quality or unhealthy will be excluded.

For all experiment grouping, data collection, and data analysis, we tried to follow the blinding roles. Data collection, data analysis, and data inputting (into excel files) were accomplished by different authors.

For experiment grouping and data collection in fertility assays, each mating cage was assigned a unique cage number without labeled with group name. Every day one author examined each cage and documented newborns, and then the data were sent to one first author (one of the four first authors). The first author inputted the daily data into the fertility assay excel file, where each cage number corresponds to a specific WT or CS female mice.

For all other experiment groupings and data collection, control or treated samples have to be clearly labeled. However, during the image taking, follicle counting, or intensity quantification, the group label for each sample was covered by a black-sticky tap and relabeled as numbers or letters. After the processes, the black-sticky taps were removed, one first author could easily find the correlation between the analyzed data and the sample information and inputted the data into the corresponding original excel file.

Before the experiment operation, each individual (oocyte, ovary, or mice) in an independent repeat or group was selected, allocated randomly and blindingly. For data collection in an independent repeat, each data point was selected randomly.

### 2.10. Statistical Analysis

All statistical graphs for western blots or DNA gels were from three independent repeats, and all statistical graphs for blood biochemical indexes were from five independent repeats. Each dot in graphs represents one repeat. If the standard error of all randomly collected individual data points in a group is significantly smaller than the average value, the corresponding sample size is appropriate and creditable. Data are presented as mean±SEM . Statistical comparisons between two groups were made with the Student’s t -test of the Excel program (Microsoft, Redmond, WA, USA). Multiple comparisons were made by using the Kruskal-Wallis one-way nonparametric ANOVA (Prism; GraphPad Software, San Diego, CA, USA). Values of p<0.05 were considered statistically significant.

## 3. Results

### 3.1. ATP5O Crotonylation Level Was the most Downregulated in the Ovaries and Plasma of CS Mice

As aforementioned, people under CS have diverse health problems, including abnormal metabolism. Moreover, these abnormalities might all exert a wide range of effects on female fertility that could negatively affect the health of the next generation. Until now, few studies have investigated whether and how PTMs, particularly new types of PTM, are correlated with abnormal metabolism under CS.

**Figure 1:**
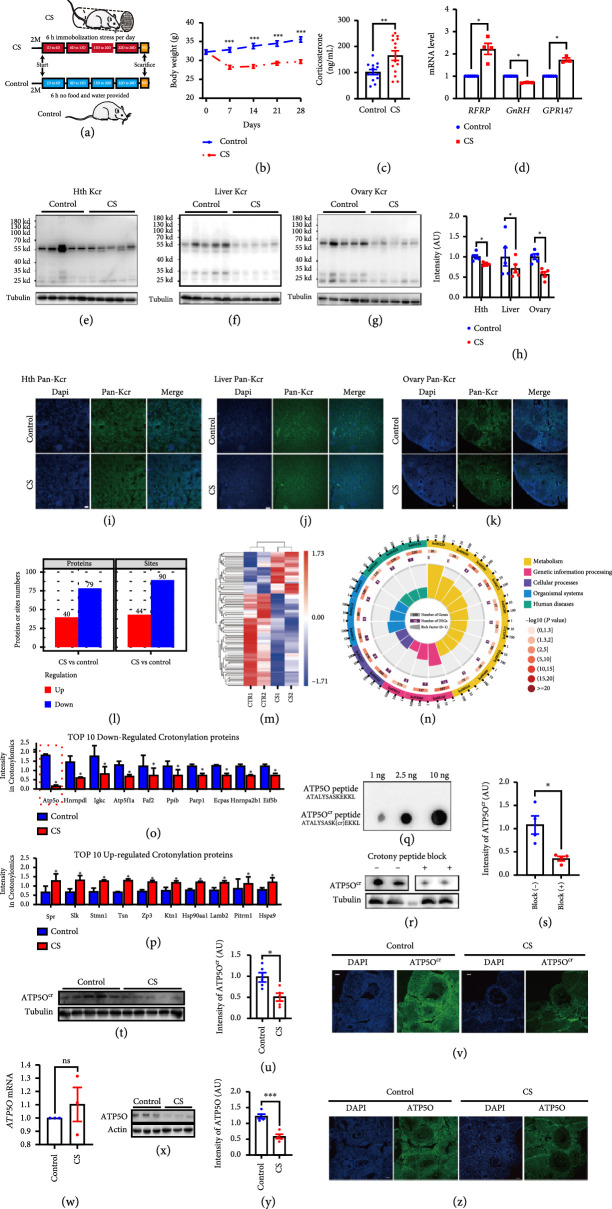
ATP5O crotonylation level was the most downregulated in the ovaries and plasma of CS mice. (a) We created a chronic stress (CS) female mouse model using a restraining cylinder. The treatment lasted for 28 days, 6 hours per day. No food and water were provided during the daily treatment. (b–d) Multiple assays indicated that the CS mouse modeling was successful. (b) The weight of the CS female mice was significantly lower than control group; (c) the serum corticosterone level of the CS female mice was significantly higher than control group; and (d) the mRNA levels of multiple HPA (hypothalamic–pituitary–adrenal) axis-related genes including RFRP (RF amide-related peptide), GnRH (gonadotropin-releasing hormone 1), and GPR147 (neuropeptide FF receptor 1), had significant differences between CS and control groups. (e–h) Crotonylation was significantly downregulated in multiple tissues of CS female mice, such as hypothalamus (Hth), livers, and ovaries. (e, h) Pan-crotonylation in hypothalamus; (f, h) pan-crotonylation in liver; (g, h) pan-crotonylation in ovary. (i, k) Pan-Kcr immunofluorescence in multiple tissues, including hypothalamus (i), livers (j), and ovaries (k), showed that pan-Kcr signal in CS group was significantly lower than in control group, whereas the morphology and condition of these tissues appeared normal in either group. Pan-Kcr in green, DNA in blue. (l, m) Quantitative crotonylomics showed that at a threshold of ≥1.3 or ≤0.77 , there were 44 upregulated and 90 downregulated crotonylation sites, with the latter covering 67.2% of all differentially crotonylation sites (DCSs). (n) Among the proteins with DCSs, metabolism-related proteins represented the largest percentage (15.97%), and there were 15 metabolism-related pathways in the top 30 KEGG pathway (50%). (o, p). Among the top 10 downregulated crotonylation sites, the crotonylation level of ATP5O at K51 decreased by about 10 folds in CS ovaries than in control ovaries. (q–h) we generated an ATP5O-K51 crotonylation-specific antibody with a crotonylated peptide ASK (crotonyl)EKKLDQVEKELL and verified the specificity through two ways. (q) Different amounts of non- and K51-crotonylated ATP5O peptide were loaded onto a dot blot PVDF membrane and detected by the ATP5O-K51 ^cr^ antibody. Only crotonylated ATP5O peptide was detected in a dose-dependent manner (Figure 1(q)). (r, s) When ATP5O-K51 ^cr^ antibody was preblocked by the crotonylated ATP5O peptide, significantly less amount of endogenous ATP5O-K51 ^cr^ was detected (Figures 1(r) and 1(s)). (t, u) Western blot by ATP5O-K51 ^cr^ antibody verified the decrement of ATP5O crotonylation in CS ovaries. (v) Immunofluorescence showed that ATP5O-K51 ^cr^ level was significantly lower in CS ovaries than in control. ATP5O-K51 ^cr^ in green, DNA in blue. (w) ATP5O mRNA level did not change in CS ovaries. (x, y) Western blot by gross ATP5O antibody showed that ATP5O level was also significantly lower in CS ovaries than in control. (z) Immunofluorescence showed that gross ATP5O level was significantly lower in CS ovaries than in control. ATP5O in green, DNA in blue. Tubulin was used as a loading control. ^*^ Indicates p<0.05 , ^**^ indicates p<0.01 , ^***^ indicates p<0.001 .

We first created a CS female mouse model according to previous studies [ 17, 31]. We analyzed body weight, plasma adrenaline (corticosterone), and HPA-related marker gene expression in the hypothalamus (Supp. table 2), and the results of these analyses indicated that the modeling was successful (Figure 1(a)– 1(d)). We found that crotonylation was significantly downregulated in multiple tissues of CS female mice, such as hypothalamus, livers, and ovaries (Figure 1(e)– 1(h)), but did not change at all in kidney (Supp. Figure 4A), heart, lung, and spleen (Supp. Figure 5A–D). Moreover, other PTMs were not changed in multiple tissues (Supp. Figures 1–4). These suggested that pan-crotonylation had close correlation with CS. We also examined the pan-PTM in the plasma of clinical females with high HAM-A (Hamilton Rating Scale for Anxiety) scores, which are widely approved to investigate the extent of CS [ 32, 33], and found that similar to the situation in CS mice, pan-crotonylation was the most downregulated in CS female plasma, whereas the other PTMs were not changed (Supp. Figure 6A–H). We also predepleted the highly rich proteins such as immunoglobulin (IgG), albumin, and fibrinogen within plasma (Supp. Figure 7A–D)) and detect pan PTMs, and found that pan-crotonylation was still the most down-regulated within CS group (Supp. Figure 7E–L). Besides, the levels of TBIL, ALT, HDL, and BUN were all significantly different (Supp. Figure 7M–R), suggesting that the health status of CS females was seriously disrupted.

The downregulated crotonylation in diverse tissues of CS mice was most likely a functional reflection because the tissue morphology and condition were similar between control and CS group, judged from pan-Kcr immunofluorescence (Figures 1(i)– 1(k)). Therefore, we decided to quantitatively analyze crotonylome. We chose the ovary for the crotonylome because it is significantly smaller than other major organs, and we could collect multiple ovaries from more individual mice (approximately 60 ovaries from 30 female mice to reach 300 mg of ovaries as one repeat); this approach effectively reduced the variation and errors from individuals (although choosing ovaries increased the workload dozens of times compared to using other organs). The results demonstrated that at a threshold of CS/control ≥ 1.3 or ≤0.77, there were 79 downregulated and 40 upregulated crotonylated proteins (Figures 1(l) and 1(m)). KEGG (Kyoto Encyclopedia of Genes and Genomes) analysis showed that many differentially crotonylated proteins (DCPs) are involved in metabolism (Figure 1(n)). Among these proteins, ATP5O, a subunit of ATP synthase, was the most downregulated (Figures 1(o) and 1(p), red dot-line rectangle labeled) at the K51 site. Then, we generated an ATP5O-K51 ^cr^-specific antibody with a crotonylated peptide ASK (crotonyl) EKKLDQVEKELL and verified the specificity through two ways. First, on a dot blot PVDF membrane, we mounted different amounts of non- and K51-crotonylated ATP5O peptide, and only crotonylated ATP5O peptide was detected by this ATP5O-K51 ^cr^ antibody in a dose-dependent manner (Figure 1(q)). Second, when we preblocked the ATP5O-K51 ^cr^ antibody with the crotonylated ATP5O peptide, significantly less amount of endogenous ATP5O-K51 ^cr^ was detected (Figures 1(r) and 1(s)). We next verified the decrement of ATP5O-K51 ^cr^ by western blotting (Figures 1(t) and 1(u)) and immunofluorescence in CS mice (Figure 1(v)). We further found that the gross protein level of ATP5O also significantly decreased in CS ovaries (Figures 1(x)– 1(z)); however, the ATP5O mRNA level did not change (Figure 1(w)). Hence, under CS, ATP5O was affected at the PTM (crotonylation) and protein levels but not the transcription level.

**Figure 2:**
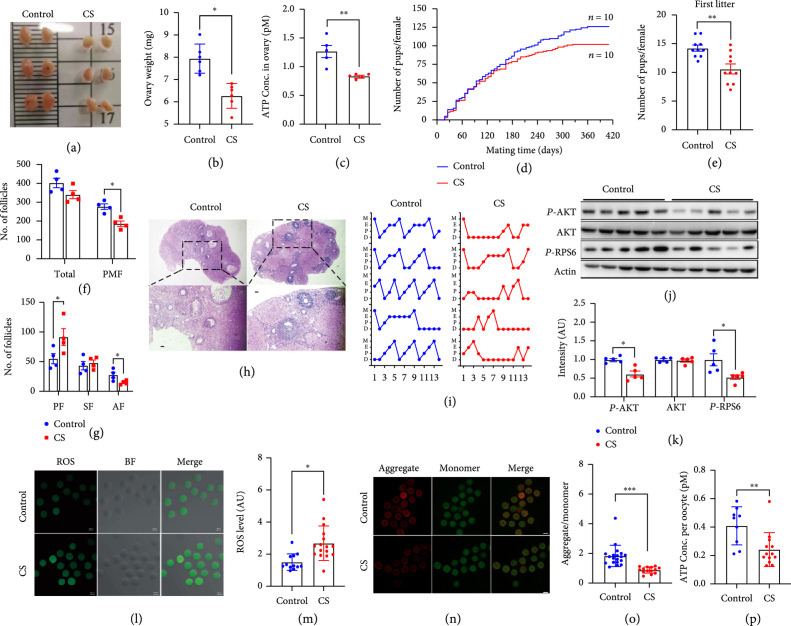
CS mice showed decreased fertility and oocyte quality. A and B. Ovary weight significantly decreased in CS female mice. C. Ovarian ATP level was significantly lower in CS group than in control. D. A 14-month-long fertility assay showed that cumulative litter size per female significantly decreased in CS female mice. E. First litter size significantly decreased in CS group. F-H. The numbers of total, primordial, and antral follicles all significantly decreased in CS group. I. The number of complete estrus cycles significantly decreased in CS group. J and K. Western blot showed that the levels of mTOR downstream kinases, p-AKT and p-RPS6, significantly decreased in CS group. L and M. ROS level significantly increased in CS group. N and O. JC1 level as indicated by the ratio of aggregate (red)/monomer (green) significantly decreased in CS group. P. ATP level significantly decreased in the oocytes of CS mice. Actin was used as a loading control. ^*^ Indicates p<0.05 , ^**^ indicates p<0.01 , ^***^ indicates p<0.001 .

Because many DCPs in CS ovaries are involved in metabolism, and abnormal metabolism affects overall health, including reproduction and, therefore, also the health of the next generation [ 34, 35], we next tested the fertility of CS female mice. We found that the ovary weight (Figures 2(a) and 2(b)), ATP level (Figure 2(c)), cumulative litter size per female (Figure 2(d)), and the first litter size (Figure 2(e)) in CS female mice all significantly decreased. Accordingly, the numbers of total, primordial, and antral follicles all significantly decreased (Figure 2(f)– 2(h)), as did the number of complete estrus cycles (Figure 2(i)). Moreover, the key regulators of the mTOR pathway—p-AKT and p-RPS6—significantly decreased in the ovaries of CS female mice (Figures 2(j) and 2(k)). Next, we found that the ROS level significantly increased (Figures 2(l) and 2(m)), whereas JC-1 and ATP levels significantly decreased (Figure 2(n)– 2(p)) in CS oocytes. All of these results suggested that the oocyte quality was impaired.

### 3.2. Phospholipid Metabolism Was Downregulated in CS Mouse Plasma

**Figure 3:**
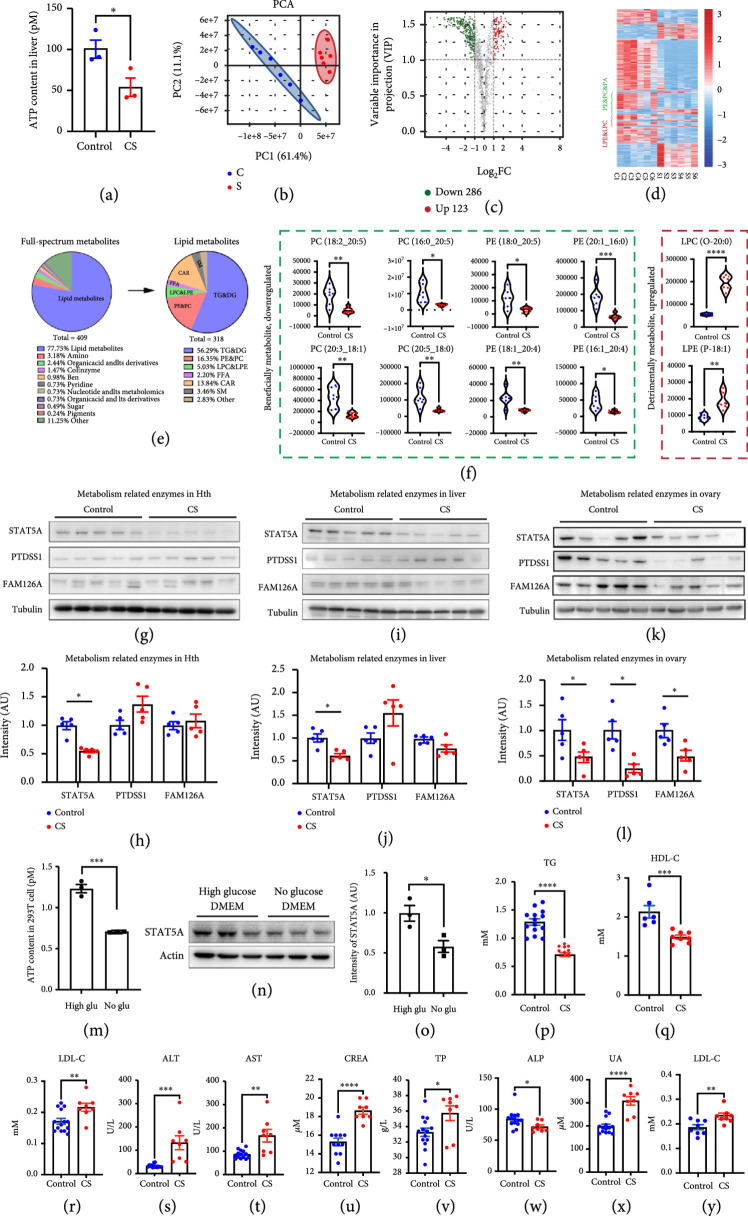
Lipid metabolism was downregulated in the plasma of CS mice. (a). Liver ATP level was significantly lowers in CS group than in control. (b). Full-spectrum metabolome was performed on the female mouse serum of CS and control groups, six repeats per group. PCA (principal component analysis) plot showed that the metabolites in CS group were fairly distinct from control group. (c, d) Volcano Plot and Heat map showed that at a threshold of CS/control ≥1.5 or ≤0.67 , there were 435 downregulated and 189 upregulated metabolites. (e) Pie charts showed differential metabolite species, 68.91% (430/624) of the differential metabolites were lipids. Among the differential lipid metabolites, the first three categories are TG (triglycerides); PC & PE & PI (phosphatidylcholine, phosphatidylethanolamine, phosphatidylinositol); and LPC & LPE (lysophosphatidylcholine, lysophosphatidylethanolamine). (f) Representative differentially downregulated PC and PE, which were beneficial for physical health, and representative differentially upregulated LPC and LPE, which were detrimental for physical health, are shown. (g–l). Western blot showed that among three lipid-metabolism-related enzymes, including STAT5A, FAM126A, and PTDSS1, STAT5A significantly decreased in the CS hypothalamus (Hth) (g, h), livers (i, j), and ovaries (k, l), whereas PTDSS1 and FAM126A significantly decreased only in CS ovaries. (m–o) Switching the 293 T cell culture media from high- to low-glucose DMEM significantly decreased cytoplasmic ATP level (m) as well as STAT5A level (n, o). (p–x) Blood biochemical index showed that multiple indexes, including TG (triglyceride), HDL-C (high-density lipoprotein cholesterol), LDL-C (low-density lipoprotein cholesterol), ALT (alanine aminotransferase), AST (aspartate aminotransferase), CREA (creatinine), TP (total protein), ALP (alkaline phosphatase), and UA (Uric Acid), were significantly different between CS and control groups. (y) Blood biochemical index assay showed that a month past CS treatment, LDL-C level in CS group was still significantly higher than control. Tubulin was used as a loading control. ^*^ indicates p<0.05 , ^**^ indicates p<0.01 , ^***^ indicates p<0.001 , ^****^ indicates p<0.0001 .

ATP5O is a subunit of ATP synthetase. Presumably, ATP5O decrement will significantly decrease the function of ATP synthetase and reduce the cytoplasmic energy level, as verified within CS oocytes (Figure 2(p)) and liver (Figure 3(a)). Because it has been reported that decreased cytoplasmic energy level could cause abnormal metabolism [ 36, 37], we sent plasma samples for full-spectrum metabolomics (Figure 3(b)). At a threshold of CS/control ≥ 1.5 or ≤0.67, there were 435 downregulated and 189 upregulated metabolites, and the downregulated metabolites covered 69.71% of all metabolites (Figures 3(c) and 3(d)). Moreover, lipids represented 68.91% of all metabolites (Figures 3(d) and 3(e)). Interestingly, several types of beneficial phospholipids, such as phosphatidylcholine (PC) and phosphatidylethanolamine (PE), comprised the second largest percentage of all lipids and significantly decreased in CS serum (Figure 3(d)– 3(f)), whereas several other types of detrimental phospholipids, such as lysophosphatidylcholine (LPC) and lysophosphatidylethanolamine (LPE), significantly increased in CS serum (Figure 3(d)– 3(f)). Next, we determined that the levels of STAT5A [ 38, 39], FAM126A [ 40] and PTDSS1 [ 41], which are important for lipid metabolism, significantly decreased in the ovary of CS female mice (Figures 3(k) and 3(l)). We also examined the levels of these three metabolic enzymes in other tissues and found that STAT5A was significantly decreased in the hypothalamus (Figures 3(g) and 3(h)) and liver (Figures 3(I) and 3(j)), but not significantly different in the kidney (Supp. Figure 8A and 8B). In support of the correlation between decreased ATP and reduced STAT5A, we switched the 293 T cell culture media from high- to low-glucose DMEM, and found the ATP level significantly decreased (Figure 3(m)), meanwhile STAT5A level also significantly reduced (Figures 3(n) and 3(o)). Finally, blood biochemical indexes, including TG, HDL-C, LDL-C, ALT, AST, CREA, TP, ALP, and UA, significantly changed (Figure 3(p)– 3(x)), among which TG, HDL-C, LDL-C, ALT, and AST are important for lipid metabolism. Notably, most indexes returned to a normal level, whereas the LDL-C level, which is critical for lipid metabolism, still significantly increased one month after CS treatment (Figure 3(y)). These results suggested that abnormal phospholipid metabolism is the primary type of metabolic abnormality in CS female mice and STAT5A might be one of the key metabolic enzymes that were significantly downregulated in various tissues.

### 3.3. Decreased Crotonylation Led to Reduced ATP5O Stability and Decreased Phospholipid Metabolism

**Figure 4:**
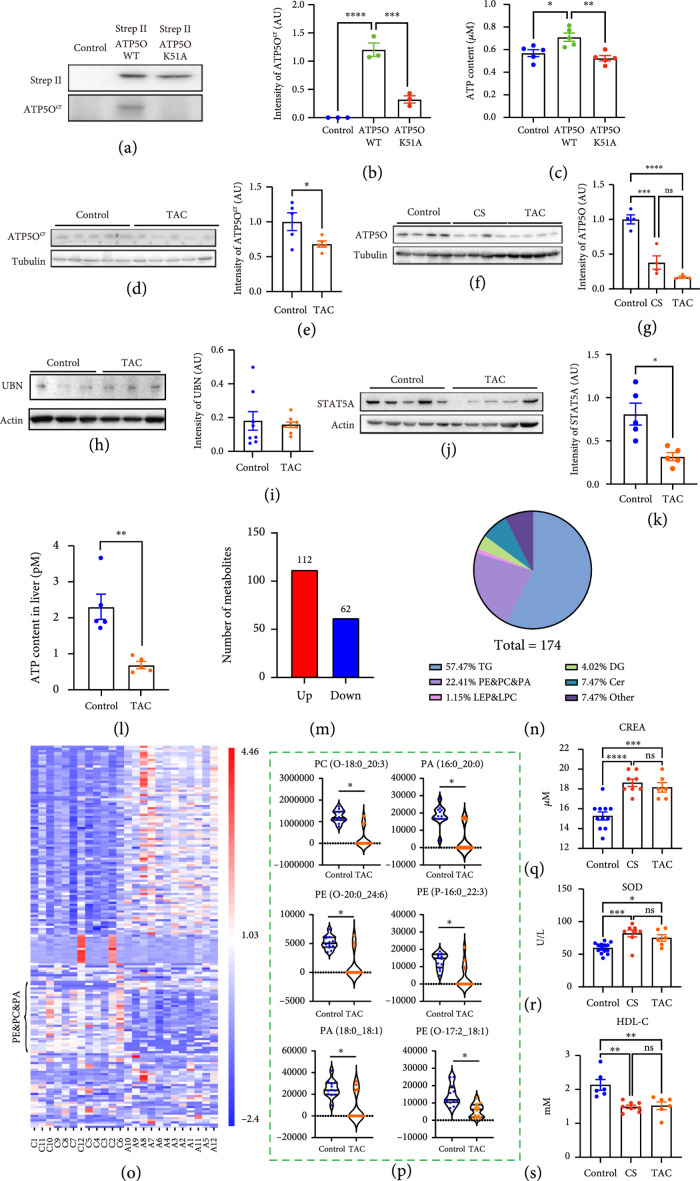
Decreased ATP5O-K51 crotonylation conduced to reduced ATP5O stability and decreased lipid metabolism. (a–c) Transfection of ATP5O-WT or K51A (inactive mutant) plasmid (in pcDNA3.1) into 293 T cells and western blot by ATP5O-K51 ^cr^ antibody showed that ATP5O-K51A had significantly decreased crotonylation level (a, b), accordingly, ATP5O K51A-transfected 293 T cells had significantly lower cytoplasmic ATP level than ATP5O-WT-transfected 293 T cells. (d–g) Western blot showed that inhibition of ATP5O crotonylation with a competitive peptide TAC (CYGRKKRRQRRRATALYSAASKEKKL) significantly reduced ATP5O-K51 crotonylation (d, e) and gross ATP5O (f, g) levels, similar to CS treatment. (h, i). A region of Ubinuclein (UBN) shared the highest similarity with TAC peptide (Supp. Figure 8); however, TAC treatment did not change UBN level at all. (j, k) Western blot showed that inhibition of ATP5O crotonylation with TAC significantly decreased STAT5A protein level. (l) TAC treatment significantly reduced liver ATP level. (m–o) Quantitative lipidome showed that at the threshold of ATP5O−TAC/Ctr≥1.5 or ≤0.67 , there were 174 differentially upregulated or downregulated lipid metabolites (DULMs or DDLMs), among this, 22.41% (39/174) was phospholipid (PE & PC & PI), and 82.05% (32/39) of phospholipid was downregulated. (p) Representative downregulated phospholipid in TAC-treated group. (q–s) Blood biochemical index assay showed that multiple indexes in TAC group, including CREA, SOD, and HDL-C, were similar to CS group and significantly different from control group. Tubulin or actin was used as a loading control. ^*^ indicates p<0.05 , ^**^ indicates p<0.01 , ^***^ indicates p<0.001 , ^****^ indicates p<0.0001 .

We guessed that crotonylation could increase the stability of ATP5O. Hence, in CS female mice, decreased ATP5O crotonylation would lead to decreased ATP5O levels. In support of this proposal, we firstly showed that non-crotonylable ATP5O mutant (K51A)-transfected 293 T cells had significantly lower cytoplasmic ATP5O crotonylation levels than ATP5O-WT-transfected 293 T cells (Figures 4(a) and 4(b)), and accordingly, the ATP level was also significantly lower in K51A-transfected cells than in WT-transfected ones (Figure 4(c)).

From the above results, CS female mice can be stated to have both decreased ATP5O levels and phospholipid metabolism. We then attempted to determine whether these factors have a causal relationship. We synthesized a peptide around the ATP5O crotonylation site fused with a membrane-penetrating TAT sequence named TAC (TAT-fused ATP5O crotonylation, CYGRKKRRQRRRATALYSAASKEKKL). In vitro assay showed that TAC can be crontonylated at K51 time-dependently by an ATP5O immunocomplex (Supp. Figures 9A and 9B), and the crotonylated TAC (TAC ^cr^) can also be decrotonylated by HDAC2 (Supp. Figures 9C and 9D), suggesting that TAC is pharmacologically active and the TAC crotonylation was reversible. Next, we injected TAC into control female mice and expected that the TAC peptide would compete with endogenous ATP5O to be crotonylated, thereby decreasing ATP5O crotonylation and ATP5O levels. We did find that TAC injection significantly decreased ATP5O crotonylation (Figures 4(d) and 4(e)) and gross ATP5O levels (Figures 4(f) and 4(g)). Blast showed that TAC sequence was highly specific (Supp. Figure 10), and even the Ubinuclein, which shared the highest similarity with TAC (Supp. Figure 10), was not changed at all by TAC inhibition (Figures 4(h) and 4(i)). Moreover, we verified that in an ATP5O-depleted cell lysate, TAC supplement did not change the pan-crotonylation level at all (Supp. Figures 11A and 11B), which further support its specificity for ATP5O-K51 ^cr^.

Next, we found that the TAC-induced ATP5O decrement also decreased STAT5A levels (Figures 4(j) and 4(k)), which was identical to the STAT5A decrement in CS hypothalamus, liver, and ovaries (Figures 3(g)– 3(l)). Besides, we found that the TAC treatment also decreased ATP levels in the liver (Figure 4(l)), which was consistent with the ATP decrement in the CS liver (Figure 3(a)).

We then attempted to determine whether inhibition of ATP5O crotonylation by TAC could largely mimic the lipid profile of stress mice. Quantitative lipidome analysis showed that at a threshold of ATP5O-TAC/Ctr ≥1.5 or ≤0.67, there were 174 differentially upregulated or downregulated lipid metabolites (DULMs or DDLMs, Figure 4(m)). Among these, 22.41% (39/174) was phospholipid (PE & PC & PI), and 82.05% (32/39) of phospholipid was downregulated (Figures 4(n)– 4(p)). Notably, the tendency of phospholipid of ATP5O-TAC/Ctr was similar to that of CS/Ctr (Figures 3(c) and 3(d)).

Moreover, the serum levels of HDL-C, CREA, and SOD in the ATP5O-inhibited group were similar to those in the CS group (Figure 4(q)– 4(s)). These results suggested that decreased ATP5O crotonylation levels might be closely correlated with downregulated phospholipid metabolism.

These results supported that decreased gross ATP5O caused by a lower level of ATP5O crotonylation is the primary factor of abnormal phospholipid metabolism.

### 3.4. Upregulated HDAC2 Phosphorylation Is Connected with Downregulated ATP5O Crotonylation in CS Mice

**Figure 5:**
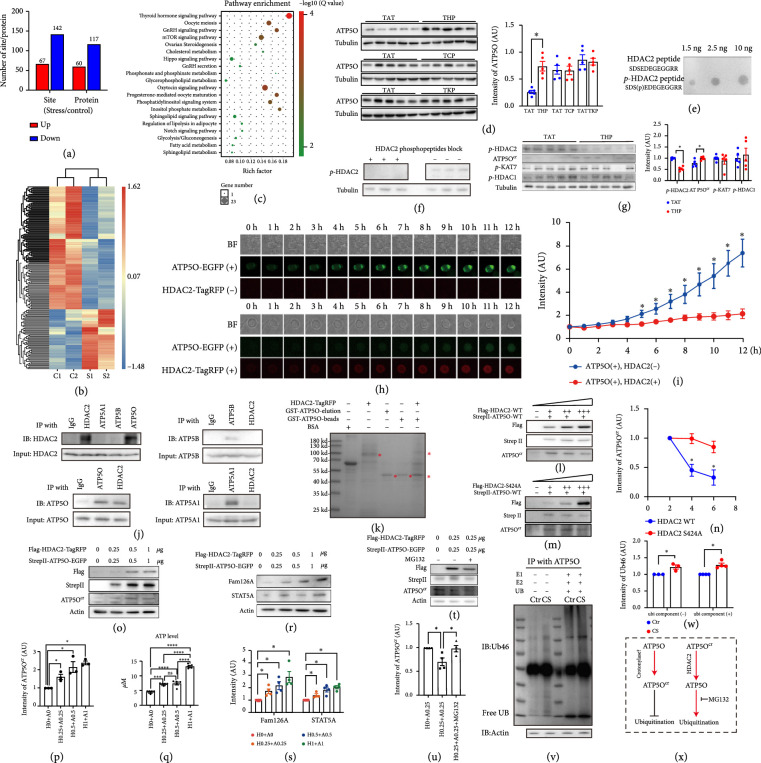
Upregulated HDAC2 phosphorylation is connected with downregulated ATP5O crotonylation in CS mice. (a, b) TMT-labeled phosphoproteomics showed that at the threshold of ≥1.2 or ≤0.833 , there are 117 downregulated and 60 upregulated phosphorylated proteins. (c) KEGG pathway showed that multiple pathways are lipid metabolism related. (d) We selected three deacetylases—HDAC2, KAT7, and CREBBP—to verify their relationship with ATP5O. Western blot showed that inhibition of HDAC2 phosphorylation at S424 with THP (CYGRKKRRQRRRSDSEDEGEGGRR) significantly decreased ATP5O, whereas inhibition of KAT7 phosphorylation at S102 with TKP (CYGRKKRRQRRRSSGSETEQVVDF) or CREBBP phosphorylation at S2361 and S2363 with TCP (CYGRKKRRQRRRQSQPPHSSPSPR) did not change ATP5O protein level at all. (e, f) We generated an ATP5O-S424 phosphorylation-specific antibody with a phosphorylated peptide SDS (phospho) EDEGEGGRR and verified the specificity through two ways. (e) Different amounts of non- and S424-phosphorylated HDAC2 peptide were loaded onto a dot blot PVDF membrane and detected by the HDAC2-S424 ^P^ antibody. Only phosphorylated HDAC2 peptide was detected in a dose-dependent manner. (f) When HDAC2-S424 ^P^ antibody was preblocked by the phosphorylated HDAC2 peptide, significantly less amount of endogenous ATP5O-K51 ^cr^ was detected. (g) Western blot showed that THP treatment significantly decreased HDAC2-S424 ^P^ (p-HDAC2) level and upregulated ATP5O-K51 ^cr^ (ATP5O ^cr^) level, whereas did not change KAT7-S102 ^P^ (p-KAT) level. Besides, THP treatment did not alter the level of HDAC1-S421 ^p^ at all, which shared the highest similarity with THP (Supp. Figure 9). (h, i) By live imaging in Sf9 cells, when only ATP5O-WT-EGFP titer was added into sf9 culture media, as time progressed, green fluorescence rapidly increased; in contrast, when both ATP5O-EGFP and HDAC2-WT-TagRFP titers were added, as time went by, the red fluorescence (HDAC2) quickly increased, whereas the increment extent of green fluorescence (ATP5O) was significantly slowed down. (j) Coimmunoprecipitation and western blot showed that HDAC2 significantly interacted with ATP5O but rarely interacted with ATP5A1 or ATP5B. (k) GST-pulldown was carried out to verify the direct binding between ATP5O and HDAC2. GST-ATP5O and his-tagged HDAC2-TagRFP were cloned, expressed, and purified in *E.coli* (From left, the fourth and fifth lane, red asterisk-labeled) and sf9 cells (From left, the third lane, red asterisk-labeled), respectively. SDS-PAGE showed that GST-ATP5O could significantly pull down HDAC2-TagRFP (From left, the sixth lane, red asterisk-labeled). L-N. Western blot showed that when a fixed amount of ATP5O-WT and increasing amounts of HDAC2-WT were cotransfected into 293 T cells, ATP5O crotonylation level rapidly decreased (l, n); in contrast, when a fixed amount of ATP5O-WT and increasing amounts of HDAC2-S424A (a nonphosphorylable mutant) were cotransfected into 293 T cells, the decrement extent of ATP5O crotonylation level became much smaller (m, n). (o–s) When HDAC2-Flag and ATP5O-StrepII were cotransfected into 293 T cells at an increasing dosage, ATP5O crotonylation (o, p) and ATP levels both increased (q); accordingly, FAM126A and STAT5A levels also increased in a dose-dependent manner (r, s). (t–u) Western blot showed that compared with ATP5O-transfected 293 T cells, HDAC2 and ATP5O cotransfected cells had decreased ATP5O crotonylation, whereas inhibition of proteasome activity with MG132 significantly recovered ATP5O crotonylation level. (v, w) Western blot showed that in an in vitro ubiquitination assay with the ATP5O-E3 ubiquitin ligase complex immunoprecipitated from CS ovaries, the ATP5O ubiquitination level was significantly higher than the level in control ovaries. X. Model: ATP5O crotonylation is resistant to ubiquitination, whereas ATP5O-K51 ^cr^ decrotonylated by HDAC2 is susceptible to ubiquitination and protease degradation. Tubulin or actin was used as a loading control. ^*^ indicates p<0.05 , ^***^ indicates p<0.001 , ^****^ indicates p<0.0001 .

It has been reported that some acetylases and deacetylases could also influence other types of PTMs. For example, histone acetyltransferase p300/CREB-binding protein-associated factor (PCAF) also affects lysine crotonylation [ 42] and isobutyrylation activity [ 43]. Next, we investigated whether the altered activities of some specific acetylases and deacetylases contributed to the downregulated ATP5O crotonylation. For this aim, we again collected ovaries and performed TMT-labeled quantitative phosphomics. We found that at a threshold of CS/control ≥1.2 or ≤0.833, there were 117 downregulated and 60 upregulated phosphorylated proteins (Figures 5(a) and 5(b)). KEGG analysis showed that multiple pathways of top 20 were involved in lipid metabolism (Figure 5(c)). Several acetylases and deacetylases with known important PTM functions were significantly more phosphorylated (Figure 5(a)– 5(c)). We selected HDAC2-Ser424, Kat7-Ser102, and CREBBP-ser2361&ser2363 for further investigations. Again, we used a TAT-fused peptide around HDAC2-Ser424 as the competing peptide (named THP, CYGRKKRRQRRRSDSEDEGEGGRR). We found that THP injection did increase gross ATP5O levels (Figure 5(d)). However, injection of TAT-fused Kat7 phosphorylated peptide (TKP, CYGRKKRRQRRRSSGSETEQVVDF) or TAT-fused CREBBP phosphorylation peptide (TCP, CYGRKKRRQRRRQSQPPHSSPSPR) failed to increase gross ATP5O levels (Figure 5(d)). These results suggested that the upregulated phosphorylation of HDAC2-S424 might promote its decrotonylation activity and contribute to ATP5O crotonylation downregulation. To support this proposal, we custom-made a HDAC2-S424 ^p^ antibody with SDS (phospho) EDEGEGGRR antigen peptide and carried out two tests to verify the specificity. First, on a dot blot PVDF membrane, we mounted different amount of non- and S424-phosphorylated HDAC2 peptide, only phosphorylated HDAC2 peptide was detected by this HDAC2-S424 ^p^ antibody in a dose-dependent manner (Figure 5(e)). Second, when we preblocked the HDAC2-S424 ^p^ antibody with the phosphorylated HDAC2 peptide; it detected significantly fewer amount of endogenous HDAC2-S424 ^p^ (Figure 5(f)). We next verified that the THP treatment significantly decreased HDAC2-S424 ^p^ and ATP5O-K51 ^cr^ but did not change KAT-S102 ^p^ (Figure 5(g)). Besides, blast showed that THP sequence was highly specific (Supp. Figure 12), and even the HDAC1-S421 ^p^, which shared the highest similarity with THP (Supp. Figure 12), was not changed at all by THP inhibition (Figure 5(g)).

From the above results, it appeared that HDAC2 activity regulates ATP5O crotonylation level. Therefore, we wanted to further analyze whether HDAC2 had specific activity against ATP5O. First, by live imaging in Sf9 cells, when we added ATP5O-WT-EGFP titer, as time progressed, green fluorescence rapidly increased; in contrast, when we added both ATP5O-EGFP and HDAC2-WT-TagRFP titers, as time went by, the red fluorescence (HDAC2) quickly increased, whereas the increment extent of green fluorescence (ATP5O) was significantly slowed down (Figures 5(h) and 5(i)). Second, coIP showed that HDAC2 apparently interacts with ATP5O but not ATP5A1 or ATP5B (Figure 5(j)). Third, we purified GST-ATP5O and HDAC2-TagRFP, and found that GST-ATP5O could significantly pull down HDAC2-TagRFP, indicating that ATP5O could directly bind HDAC2 (Figure 5(k)). Fourth, when we co-transfected a fixed amount of ATP5O-WT and increasing amounts of HDAC2-WT into 293 T cells, ATP5O crotonylation level rapidly decreased (Figures 5(l) and 5(n)); in contrast, when we cotransfected a fixed amount of ATP5O-WT and increasing amounts of HDAC2-S424A (a nonphosphorylable mutant) into 293 T cells, the decrement extent of ATP5O crotonylation level became much smaller (Figures 5(m) and 5(n)). These results indicated that upregulated HDAC2 phosphorylation at S424 could specifically decrotonylate ATP5O at K51.

We then wanted to investigate whether the decrotonylation capability of HDAC2 could induce dysregulated phospholipid metabolism independently of ATP5O. If this were to be true, we would expect that even as ATP5O increased, HDAC2 could cause decreased levels of the essential lipid metabolic enzymes shown above. However, we observed the opposite results: In the premise of increased HDAC2, as ATP5O increased, both ATP5O crotonylation and ATP levels increased (Figure 5(o)– 5(q)), accordingly FAM126A and STAT5A also significantly increased (Figures 5(r) and 5(s)). This result further indicates that HDAC2 decrease phospholipid metabolism through downregulating ATP5O and ATP level.

Next, we wished to determine whether decreased ATP5O crotonylation could result in decreased ATP5O level. The results showed that compared with ATP5O-transfected cells, HDAC2 and ATP5O cotransfected cells decreased ATP5O crotonylation level, whereas inhibition of proteasome activity with MG132 significantly increased ATP5O crotonylation levels (Figures 5(t) and 5(u)). Accordingly, in an in vitro ubiquitination assay with the ATP5O-E3 ubiquitin ligase complex immunoprecipitated from CS ovaries, the ATP5O ubiquitination level was significantly higher than the level in control ovaries (Figures 5(v) and 5(w)).

These results indicated that decreased ATP5O level is essential for the reduced phospholipid metabolism, and HDAC2 activity is critical for the ATP5O crotonylation and gross ATP5O levels (Figure 5(x)).

### 3.5. Correcting HDAC2 Phosphorylation Rescued Abnormal Phospholipid Metabolism

**Figure 6:**
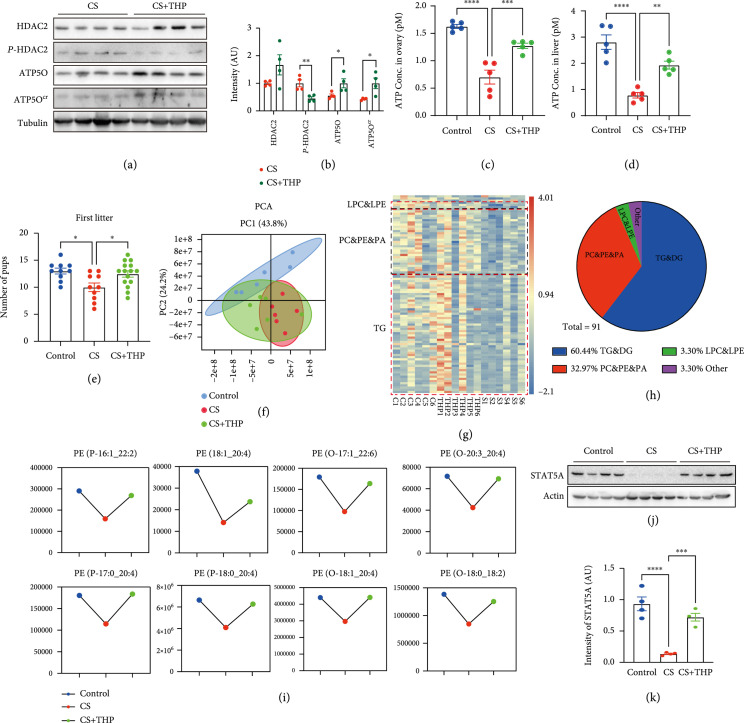
Correcting HDAC2 phosphorylation rescued abnormal lipid metabolism. (a, b) Western blot showed that inhibition of HDAC2 activity (phosphorylation) by a peptide THP (CYGRKKRRQRRRSDSEDEGEGGRR) significantly reduced the level of p-HDAC2, whereas ATP5O crotonylation and gross ATP5O levels significantly increased. (c, d) ATP levels in CS ovaries (c) and livers (d) were significantly lower than control, whereas THP treatment significantly recovers the decreased ATP levels close to control. (e) Mating test showed that the first litter size was significantly lower than control, while THP injection recovered the decreased litter size close to control. (f–h) Lipid metabolomes were analyzed for the female mouse serum of control, CS, and CS+THP group; six repeats per group. PCA plot showed that the metabolites in the CS group are completely separate from control group, with the CS+THP group partially overlapping with the control group (f). Heat map showed that at a threshold of CS+THP vs. CS≥1.5 or ≤0.67 , there were 91 differentially upregulated or downregulated lipid metabolites (DULMs or DDLMs) that were recovered close to the control (g). And the beneficial lipid form—PE, PA, and PC—covered the second largest percentage (30 of 91, 32.97%) (g, h). (i) Representative differentially downregulated PEs, which were beneficial for physical health, are shown. (j, k) Western blot showed that STAT5A level significantly decreased in CS group, whereas THP treatment significantly recovered the STAT5A level of CS group close to control group. Tubulin was used as a loading control. ^*^ indicates p<0.05 , ^**^ indicates p<0.01 , ^***^ indicates p<0.001 , ^****^ indicates p<0.0001 .

From the above findings, upon activation (phosphorylation), it can be stated that HDAC2 directly decrotonylates ATP5O and decreases its stability, and ATP5O decrement led to downregulated lipid metabolism. Therefore, we guessed that target-correcting HDAC2 phosphorylation could rescue abnormal lipid metabolism in CS female mice. We injected THP into CS mice and found that p-HDAC2 significantly decreased, whereas both ATP5O crotonylation and gross ATP5O levels both significantly increased (Figures 6(a) and 6(b)). Furthermore, THP injection also recovered the decreased ATP level close to that of the control group in ovaries (Figure 6(c)) and liver (Figure 6(d)). Moreover, THP injection also recovered the litter size in the CS group close to that of the control group (Figure 6(e)). Next, we analyzed the lipid metabolome of the control, CS, and CS+THP groups in parallel. A PCA plot showed that before THP treatment, the lipid profiles in CS serum were completely separated from control serum, whereas after THP treatment, the lipid profiles in CS serum partially overlapped with the control group (Figure 6(f)). A heat map demonstrated that at a threshold of CS + THP vs. CS ≥1.5 or ≤0.67, there were 91 lipid metabolites that were recovered close to the control, particularly the beneficial phospholipid form—PE & PA & PC—which decreased in the CS group and recovered in the CS+THP group, comprising the second-largest percentage (30 of 91, 32.97%) (Figures 6(g)– 6(i)). Finally, THP injection also rescued the decreased STAT5A level in the stress group close to that of the control (Figures 6(j) and 6(k)).

These results indicated that inhibition of HDAC2 activity could partially rescue abnormal lipid metabolism by correcting ATP5O crotonylation and gross ATP5O level.

### 3.6. Clinical Female Patients with High stress Scores Have Decreased ATP5O as Well as Abnormal Phospholipid Metabolism Overlapping with CS Mice

**Figure 7:**
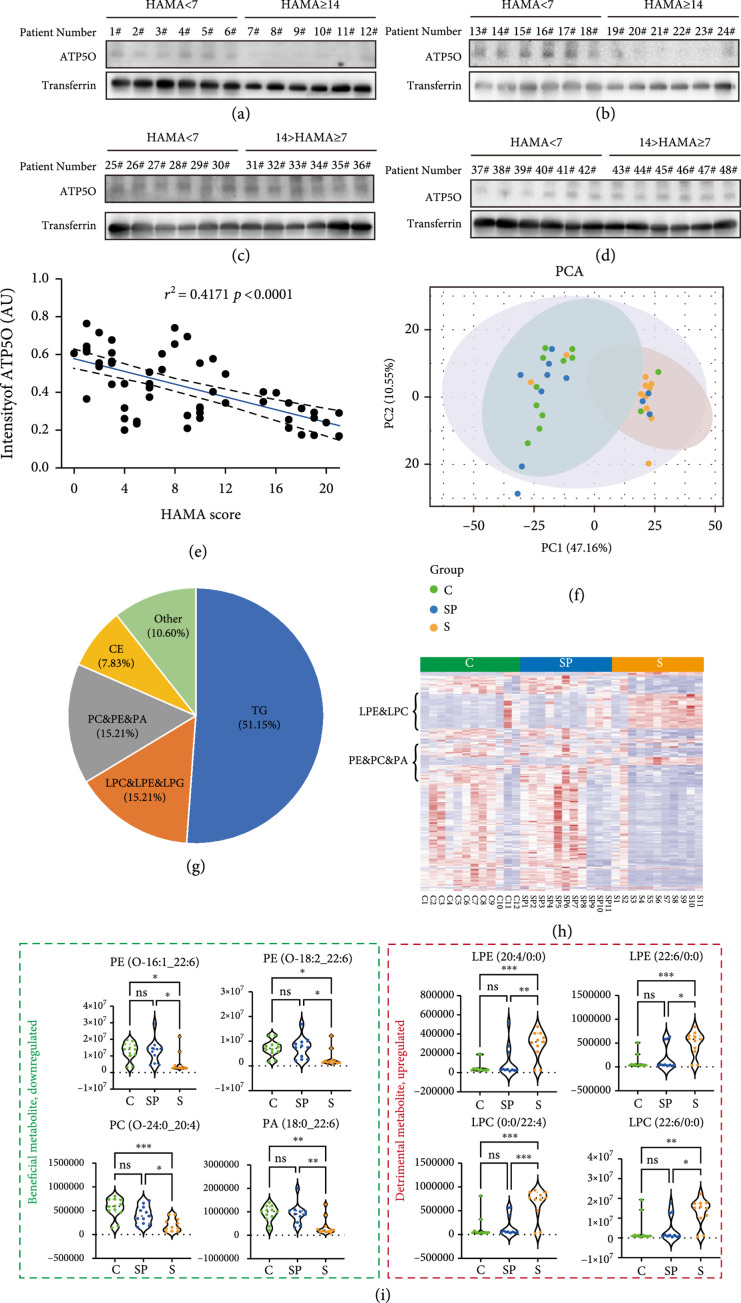
Clinical female patients with high stress scores have decreased ATP5O as well as abnormal phospholipid metabolism overlapping with CS mice. (a–d) Western blots showed that the ATP5O level in the stress-prone ( 7≤HAMA score≤14 ) group was not significantly different from control group ( HAMA score≤7 ), but ATP5O levels in the stress group ( HAMA score≥14 ) were significantly lower than control group. (e) Quantification of ATP5O level showed that ATP5O level was negatively correlated with HAMA score. (f–h) Lipid metabolomes were determined for the human female serum of control, stress-prone, and stress groups, with ten repeats per group. Heat map of differentially upregulated or downregulated lipid metabolites (DULMs or DDLMs) showed that at a threshold of stress/control ≥1.5 or ≤0.67 , there were 62 upregulated and 155 downregulated lipid metabolites. Among these, 15.207% (33/217) was phospholipids (PE & PC & PI) and 15.207% (33/217) was lysophospholipids (LPE & LPC & LPA). Furthermore, 36.37% (12/33) of phospholipid, which are beneficial, was downregulated; and 100% (33/33) of lysophospholipids, which are detrimental, was upregulated. (i) Representative beneficial PE & PC & PA and detrimental LPE & LPC are shown. Transferrin was used as a loading control. ^*^ indicates p<0.05 , ^**^ indicates p<0.01 , ^***^ indicates p<0.001 .

Finally, we attempted to see whether the correlation between the stress scores, the level of ATP5O-K51 ^cr^ & gross ATP5O, and the phospholipid metabolism in clinical CS females was similar to that in CS mice. Unfortunately, we were unable to detect ATP5O-K51 ^cr^ in clinical female plasma, the reason was probably that the antigen sequence (the corresponding crotonylated sequence was used for ATP5O-K51 ^cr^ antibody) in mouse ATP5O was somewhat different from the corresponding sequence in human ATP5O (Supp. Figure 13, red dot line-boxed). Therefore, we had to thoroughly examine the level of gross ATP5O in the plasma of control or chronic stress patients. We found that although the ATP5O level in the stress-prone group (14 > HAMA ≥7) was not significantly different from the control group (HAMA <7) (Figures 7(c) and 7(d)), the ATP5O level in the stress group (HAMA ≥14) was significantly lower than the control group (Figures 7(a) and 7(b)). Overall, the HAMA score and gross ATP5O level were negatively correlated (Figure 7(e)).

We next examined the lipid profile of human serum. We found that the plasma of the stress group had a lipid profile that partially overlapped with that in CS female mice (Figure 7(f)). In particular, the heat map of differentially upregulated or downregulated lipid metabolites (DULMs or DDLMs) showed that at a threshold of stress vs. control ≥1.5 or ≤0.67, there were 62 upregulated and 155 downregulated lipid metabolites. Among these, 15.207% (33/217) was phospholipids (PE & PC & PI) and 15.207% (33/217) was lysophospholipids (LPE & LPC & LPA). Notably, 36.37% (12/33) of phospholipid, which are beneficial, was downregulated; and 100% (33/33) of lysophospholipids, which are detrimental, was upregulated (Figure 7(g)– 7(i)). The differences between humans and mice were largely similar.

These results suggested that downregulated ATP5O protein level, together with abnormal lipid profile, particularly downregulated PE & PA & PC and upregulated LPE & LPA, with relatively good overlapping with the situation in CS female mice.

## 4. Discussion

In the present study, we, for the first time, have revealed a critical link between abnormal lipid metabolism and altered PTM levels of diverse proteins in CS model female mice and clinical females. Furthermore, we have partially rescued the lipid metabolic abnormality by target-correcting the abnormal PTM.

PTMs directly regulate the activities and stability of diverse key enzymes and proteins, and abnormal PTMs are the major cause of many nonhereditary chronic diseases. However, little is known about whether ATP synthase, the key complex to generate ATP, could be regulated by PTMs. In this study, we have, for the first time, shown that the Lys51 crotonylation of ATP5O, one of the F1 subunits, was downregulated the most. We also verified that crotonylation could stabilize ATP5O, thus decreasing crotonylation through a crotonylation-competitive peptide, leading to decreased gross ATP5O levels and causing the reduction of STAT5A, which has been shown to regulate lipid metabolism [ 38, 39]. The key function of the ATP synthase complex is to generate ATP at the mitochondria inner membrane [ 44]. Dysfunction of ATP synthase, mutation of synthase subunits, and abnormal epigenetic modification of ATP synthase genes are associated with severe human diseases, cancer development, and drug resistance [ 45]. Comprehensibly, the reduction of any ATP synthase subunit will decrease the activity of the entire ATP synthase and thereby lower ATP levels. In our study, the ATP5O level significantly reduced and, accordingly, ATP levels also significantly decreased under CS. The correlation of reduced ATP level with abnormal metabolism has been reported [ 36, 37, 46]; however, details regarding how the metabolism is altered are still lacking. Our quantitative crotonyl-omics demonstrated that the crotonylation level of ATP5O decreased the most and much more than another ATP synthase subunit, Atp5f1a, indicating that the decrotonylation activity of HDAC2 has good preference for ATP5O. CoIP showed that HDAC2 had clear interactions with ATP5O but not with ATP5A1 or ATP5B, suggesting that ATP5O has more freedom than other ATP synthase subunits. Hence, the activity of ATP synthase can be efficiently and easily adjusted through the regulation of a single subunit. There are other examples illustrating that a special subunit in a complex has more freedom and is involved in more diverse processes than other subunits; for example, RPS6 of the ribosomal complex [ 47] and CENP-A of the centromeric protein complex [ 48].

HDAC2 is a well-known deacetylase. However, several recent studies have shown that HDAC2 also has decrotonylation activity involved in diverse cellular processes [ 49, 50]. In the present study, we found that HDAC2 phosphorylation at Ser424 was upregulated under CS and was responsible for ATP5O decrotonylation, and purified HDAC2 could decrotonylate purified ATP5O *in vivo* as well. We also demonstrated that the downregulation of p-HDAC2 could partially recover ATP5O levels and the abnormal lipid metabolism under CS. Such evidence suggested that HDAC2 is the primary PTM enzyme to decrotonylate ATP5O, and the HDAC2-ATP5O PTM chain is the main factor that leads to the abnormal lipid metabolism under CS. Furthermore, because the entire sequence and the PTM sites of ATP5O and HDAC2 are highly conserved between humans and mice, THP might therefore be a therapeutic peptide against female CS.

Phosphatidylcholine, phosphatidylethanolamine, and phosphatidylinositol are the major components of the cell membrane or organelle membrane, and they contribute to proliferation, apoptosis, organelle membrane fusion, oxidative phosphorylation, mitochondrial biogenesis, and autophagy. Alteration in their contents and dynamics is associated with diverse degenerating diseases and metabolic disorders [ 51– 56]. In the present study, CS caused a significant decrease of PE & PC & PA in both CS mice and stressed humans, whereas inhibition of HDAC2 phosphorylation partially recovered the levels of these lipids close to the control, suggesting that correcting the HDAC2-ATP5O PTM chain could significantly rescue the abnormal lipid metabolism in CS females. Interestingly, it is reported that the hippocampus lipidome of CS rats showed a decrease in PE&PC&PA and an increase in detrimental LPE&LPC [ 17], largely agreeing with our findings.

**Figure 8:**
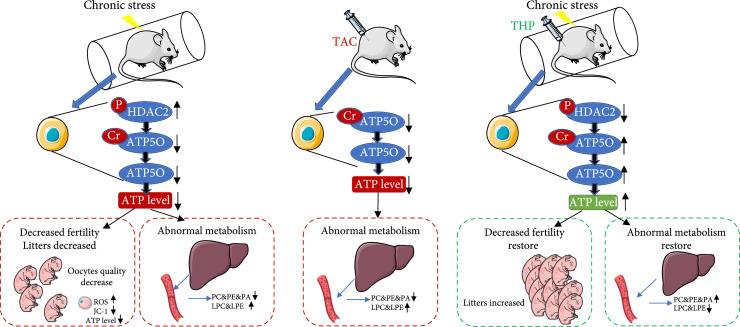
Model: chronic stress caused aberrant level of two types of PTMs, which reduce cytoplasmic ATP level and consequently reduced beneficial phospholipids. Left: Under chronic stress (CS), HDAC2-S424 ^p^ was upregulated, which resulted in downregulated ATP5O-K51 ^cr^. Downregulated ATP5O-K51 ^cr^ consequently conduced to reduced gross ATP5O level, which finally caused decreased cytoplasmic ATP level and significantly decreased oocyte quality, fertility, and beneficial phospholipids. Middle: TAC is a competitive peptide that was able to specifically downregulate ATP5O-K51 ^cr^. Downregulated ATP5O-K51 ^cr^ consequently conduced to reduced gross ATP5O level and cytoplasmic ATP level, which finally significantly decreased oocyte quality, fertility, and beneficial phospholipids. Therefore, TAC treatment could partially recapitulate the decreased fertility and phospholipid in CS mice. Right: THP is a competitive peptide that was able to specifically downregulate the abnormally high level of HDAC2-S424 ^p^ in CS mice, which in turn recovered downregulated ATP5O-K51 ^cr^, gross ATP5O level, and cytoplasmic ATP level close to control. Recovered ATP level finally recovered oocyte quality, fertility, and phospholipid metabolism in CS mice, Therefore, THP treatment could partially rescue the decreased fertility and phospholipid in CS mice.

In all, we found that plasma ATP5O or p-HDAC2 could be novel markers for the abnormal metabolism in CS model mice or clinical patients. p-HDAC2 upregulation reduced ATP5O crotonylation, destabilized ATP5O, and led to decreased gross ATP5O levels. Target-inhibiting HDAC2 phosphorylation could upregulate ATP5O crotonylation, destabilize ATP5O, and thereby recover gross ATP5O levels, thus rescuing the abnormal metabolism in CS model female mice (Figure 8). Further studies are required to investigate how these incidences occur and are regulated in detail.

## Data Availability

The data that support the findings of this study are available from the corresponding author upon reasonable request. Supplementary datasets 1-5 have been deposited into Zendo ( https://zenodo.org/record/5559428#.YWLAEPkt2Uk). The DOI is doi: 10.5281/zenodo.5559428. Raw data and extracted text files for quantitative phosphoproteomics and crotonylproteomics have been deposited into PRIDE, the accession No. is PXD028932 and PXD028961, respectively.

## Abbreviations

AD: Alzheimer’s disease
AF: Antral follicles
AKT: Thymoma viral proto-oncogene 1
ALP: Alkaline phosphatase
ALT: Alanine aminotransferase
APO: Apoprotein
AST: Aspartate aminotransferase
ATP5A1: ATP synthase, H+ transporting, mitochondrial F1 complex, alpha subunit 1
ATP5B: ATP synthase, H+ transporting mitochondrial F1 complex, beta subunit
ATP5O: ATP synthase, H+ transporting, mitochondrial F1 complex, O subunit
BUN: Blood urea nitrogen
CDYL: Chromodomain Y-like protein
CEPT1: Choline-ethanolamine phosphotransferase 1
CS: Chronic stress
DCPs: Differentially Crotonylated proteins
dhSM: Dihydrosphingomyelin
DDLMs: Differentially downregulated lipid metabolites
DDMs: Downregulated metabolites
DULM: Differentially upregulated lipid metabolites
FAM126A: Family with sequence similarity 126 member A
GnRH: Gonadotropin releasing hormone
GPR147: Neuropeptide FF receptor 1
HARC: Hamilton anxiety rating scale
HAM-A: Hamilton rating scale for anxiety
HDAC2: Histone deacetylase 2
HDL: High-density lipoprotein
HPA axis: Hypothalamic–pituitary–adrenal axis
IHTG: Intrahepatic-triglycerides
KEGG: Kyoto Encyclopedia of Genes and Genomes
LDL-C: Low-density lipoprotein cholesterol
LPA: Lysophosphatidic acid
LPC: Lysophosphatidylcholine
LPE: Lysophosphatidylethanolamine
NSCLC: Non-small-cell lung cancer
PA: Phosphatidic acid
PC: Phosphatidylcholine
PCAF: P300/CREB-binding protein-associated factor
PCA plot: Principal component analysis plot
PE: Phosphatidylethanolamine
PF: Primary follicles
PMF: Primordial follicle
PPAR *α*: Peroxisome proliferator-activated receptor *α*
PTDSS1: Phosphatidylserine synthase 1
PTM: Protein post-translational modification
RFRP: RF amide-related peptide
ROS: Reactive oxygen species
RPS6: Ribosomal protein S6
SCLC: Small-cell lung cancer
SF: Secondary follicles
SM: Sphingomyelin
SP: Stress-prone group
STAT5A: Signal transducer and activator of transcription 5A
TAC: TAT-fused ATP5O crotonylation sequence
TBIL: Total bilirubin
TCP: TAT-fused CREBBP phosphorylation peptide
TF: Total follicles
TG: Triglyceride
THP: TAT-fused HDAC2 phosphorylation peptide
TKP: TAT-fused Kat7 phosphorylation peptide
TMT-labeled: Tandem mass tag labeled
TP: Total protein
UA: Uric acid
VLDL-TG: Very-low-density lipoprotein-triglycerides.

## Supplementary Materials

Supplementary Materials Supplementary figure 1: pan-crotonylation level was the most downregulated in the hypothalamus of CS mice. Supplementary figure 2: pan-crotonylation level was the most downregulated in the liver of CS mice Supplementary figure 3: pan-crotonylation level was the most downregulated in the ovary of CS mice. Supplementary figure 4: there were no significant differences for various pan-PTMs in the kidney. Supplementary figure 5: there were no significant differences for pan-crotonylation in various tissues. Supplementary figure 6: plasma of clinical females with high HAM-A scores has downregulated crotonylation. Supplementary figure 7: pan-crotonylation was still the most downregulated in preabsorbed plasma of clinical females with high HAM-A scores. Supplementary figure 8: there were no significant differences for the lipid metabolism-related enzymes in the kidneys. Supplementary figure 9: TAC is pharmacologically active and the TAC crotonylation was reversible. Supplementary figure 10: TAC sequence is highly specific for ATP5O. Supplementary figure 11: TAC is highly specific for ATP5O-K51cr. Supplementary figure 12: THP sequence is highly specific for HDAC2. Supplementary figure 13: protein sequence alignment between mouse and human ATP5O. Supplementary dataset 1, Supplementary dataset 2, Supplementary dataset 3, Supplementary dataset 4, Supplementary dataset 5, and Supplementary dataset 6. Supplementary table 1: radom mating table Supplementary table 2. Primer for qPCR Supplementary table 3. Plasmid construction primers for mouse HDAC2 and ATP5O Supplementary table 4. Mutagenesis primers of HDAC2 and ATP5O Supplementary table 5. In-vitro ubiquitination reaction components.
