# Supplementary figures and images for: ATP5O Hypo-crotonylation Caused by HDAC2 Hyper-Phosphorylation Is a Primary Detrimental Factor for Downregulated Phospholipid Metabolism under Chronic Stress
Source: Research (Wash D C). 2022 Nov 24;2022:9834963. doi: 10.34133/2022/9834963 (PMC11030818; doi:10.34133/2022/9834963)

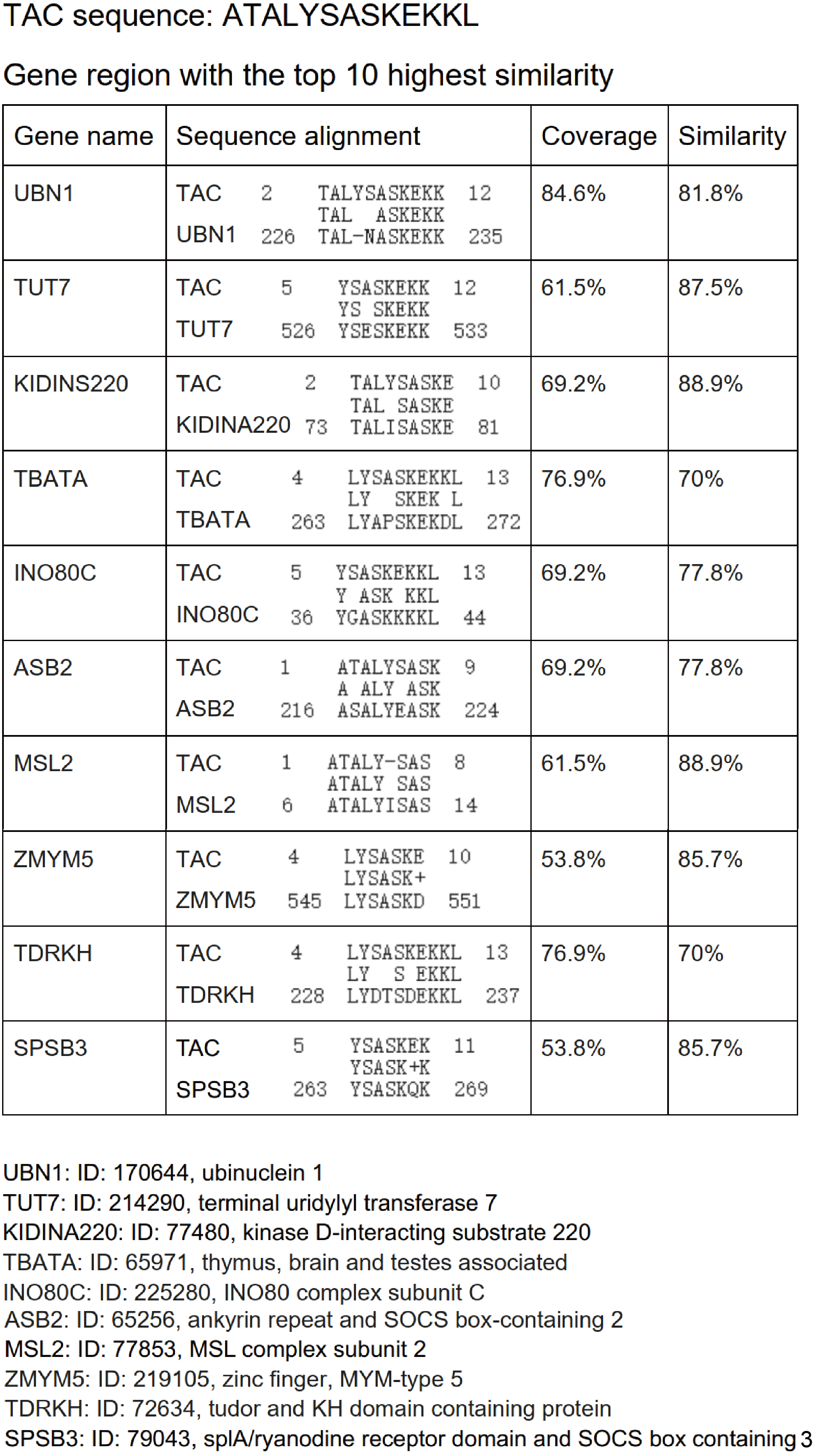

Supplement: Supplementary Materials — Supplementary figure 1: pan-crotonylation level was the most downregulated in the hypothalamus of CS mice. Supplementary figure 2: pan-crotonylation level was the most downregulated in the liver of CS mice Supplementary figure 3: pan-crotonylation level was the most downregulated in the ovary of CS mice. Supplementary figure 4: there were no significant differences for various pan-PTMs in the kidney. Supplementary figure 5: there were no significant differences for pan-crotonylation in various tissues. Supplementary figure 6: plasma of clinical females with high HAM-A scores has downregulated crotonylation. Supplementary figure 7: pan-crotonylation was still the most downregulated in preabsorbed plasma of clinical females with high HAM-A scores. Supplementary figure 8: there were no significant differences for the lipid metabolism-related enzymes in the kidneys. Supplementary figure 9: TAC is pharmacologically active and the TAC crotonylation was reversible. Supplementary figure 10: TAC sequence is highly specific for ATP5O. Supplementary figure 11: TAC is highly specific for ATP5O-K51cr. Supplementary figure 12: THP sequence is highly specific for HDAC2. Supplementary figure 13: protein sequence alignment between mouse and human ATP5O. Supplementary dataset 1, Supplementary dataset 2, Supplementary dataset 3, Supplementary dataset 4, Supplementary dataset 5, and Supplementary dataset 6. Supplementary table 1: radom mating table Supplementary table 2. Primer for qPCR Supplementary table 3. Plasmid construction primers for mouse HDAC2 and ATP5O Supplementary table 4. Mutagenesis primers of HDAC2 and ATP5O Supplementary table 5. In-vitro ubiquitination reaction components. [file 9834963.f1.zip › Supplementary figure 10.jpg]

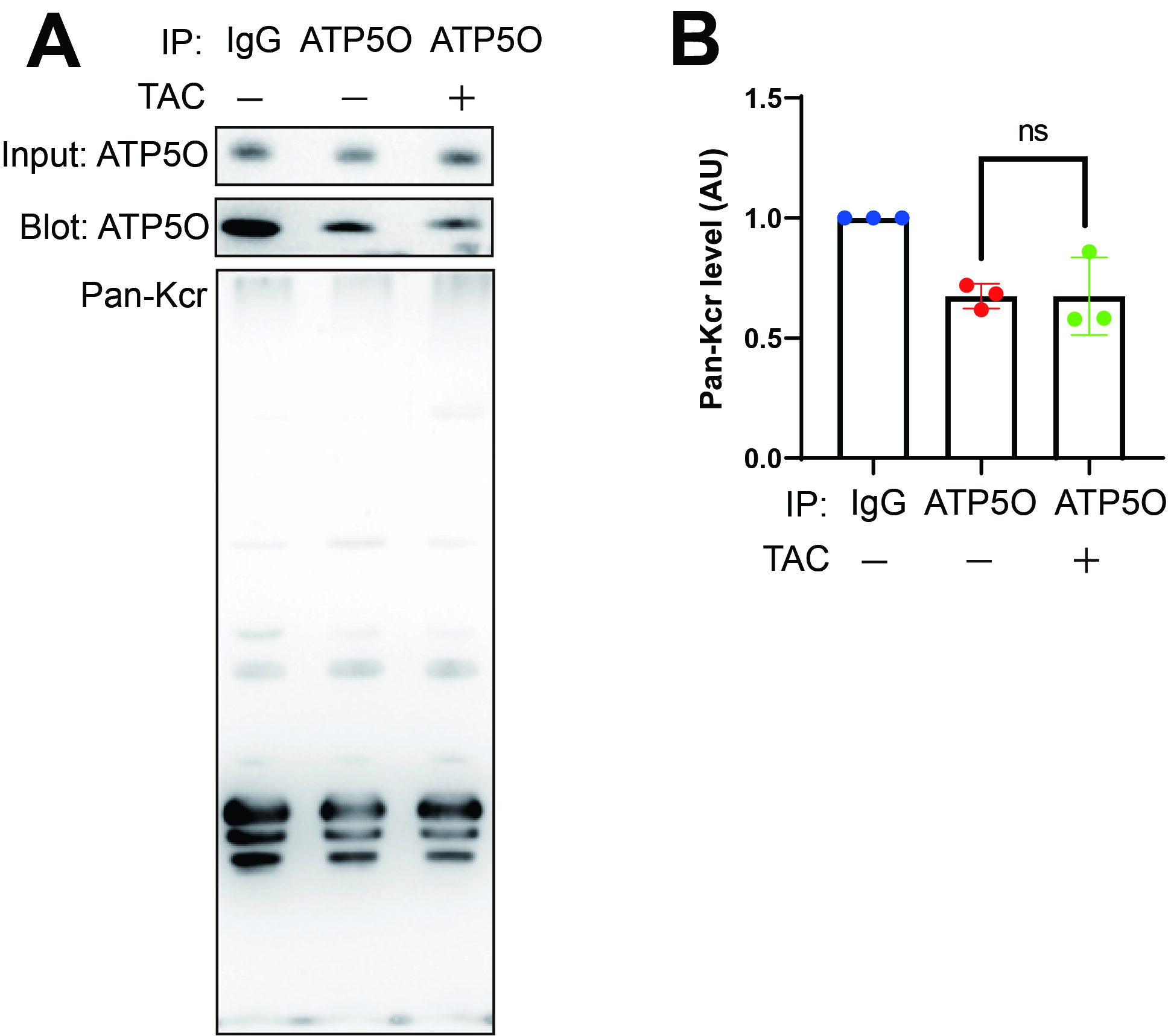

Supplement: Supplementary Materials — Supplementary figure 1: pan-crotonylation level was the most downregulated in the hypothalamus of CS mice. Supplementary figure 2: pan-crotonylation level was the most downregulated in the liver of CS mice Supplementary figure 3: pan-crotonylation level was the most downregulated in the ovary of CS mice. Supplementary figure 4: there were no significant differences for various pan-PTMs in the kidney. Supplementary figure 5: there were no significant differences for pan-crotonylation in various tissues. Supplementary figure 6: plasma of clinical females with high HAM-A scores has downregulated crotonylation. Supplementary figure 7: pan-crotonylation was still the most downregulated in preabsorbed plasma of clinical females with high HAM-A scores. Supplementary figure 8: there were no significant differences for the lipid metabolism-related enzymes in the kidneys. Supplementary figure 9: TAC is pharmacologically active and the TAC crotonylation was reversible. Supplementary figure 10: TAC sequence is highly specific for ATP5O. Supplementary figure 11: TAC is highly specific for ATP5O-K51cr. Supplementary figure 12: THP sequence is highly specific for HDAC2. Supplementary figure 13: protein sequence alignment between mouse and human ATP5O. Supplementary dataset 1, Supplementary dataset 2, Supplementary dataset 3, Supplementary dataset 4, Supplementary dataset 5, and Supplementary dataset 6. Supplementary table 1: radom mating table Supplementary table 2. Primer for qPCR Supplementary table 3. Plasmid construction primers for mouse HDAC2 and ATP5O Supplementary table 4. Mutagenesis primers of HDAC2 and ATP5O Supplementary table 5. In-vitro ubiquitination reaction components. [file 9834963.f1.zip › Supplementary figure 11.jpg]

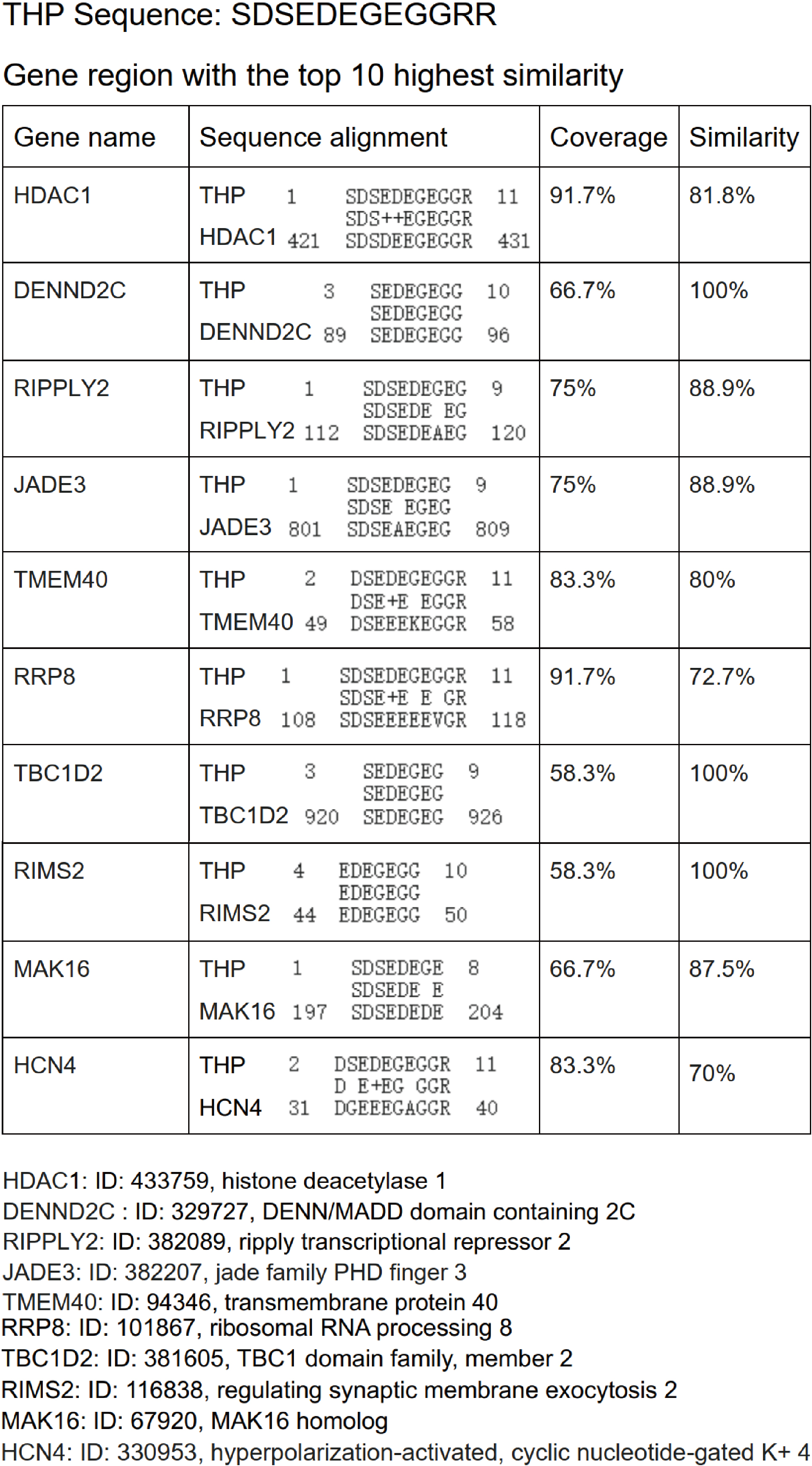

Supplement: Supplementary Materials — Supplementary figure 1: pan-crotonylation level was the most downregulated in the hypothalamus of CS mice. Supplementary figure 2: pan-crotonylation level was the most downregulated in the liver of CS mice Supplementary figure 3: pan-crotonylation level was the most downregulated in the ovary of CS mice. Supplementary figure 4: there were no significant differences for various pan-PTMs in the kidney. Supplementary figure 5: there were no significant differences for pan-crotonylation in various tissues. Supplementary figure 6: plasma of clinical females with high HAM-A scores has downregulated crotonylation. Supplementary figure 7: pan-crotonylation was still the most downregulated in preabsorbed plasma of clinical females with high HAM-A scores. Supplementary figure 8: there were no significant differences for the lipid metabolism-related enzymes in the kidneys. Supplementary figure 9: TAC is pharmacologically active and the TAC crotonylation was reversible. Supplementary figure 10: TAC sequence is highly specific for ATP5O. Supplementary figure 11: TAC is highly specific for ATP5O-K51cr. Supplementary figure 12: THP sequence is highly specific for HDAC2. Supplementary figure 13: protein sequence alignment between mouse and human ATP5O. Supplementary dataset 1, Supplementary dataset 2, Supplementary dataset 3, Supplementary dataset 4, Supplementary dataset 5, and Supplementary dataset 6. Supplementary table 1: radom mating table Supplementary table 2. Primer for qPCR Supplementary table 3. Plasmid construction primers for mouse HDAC2 and ATP5O Supplementary table 4. Mutagenesis primers of HDAC2 and ATP5O Supplementary table 5. In-vitro ubiquitination reaction components. [file 9834963.f1.zip › Supplementary figure 12.jpg]

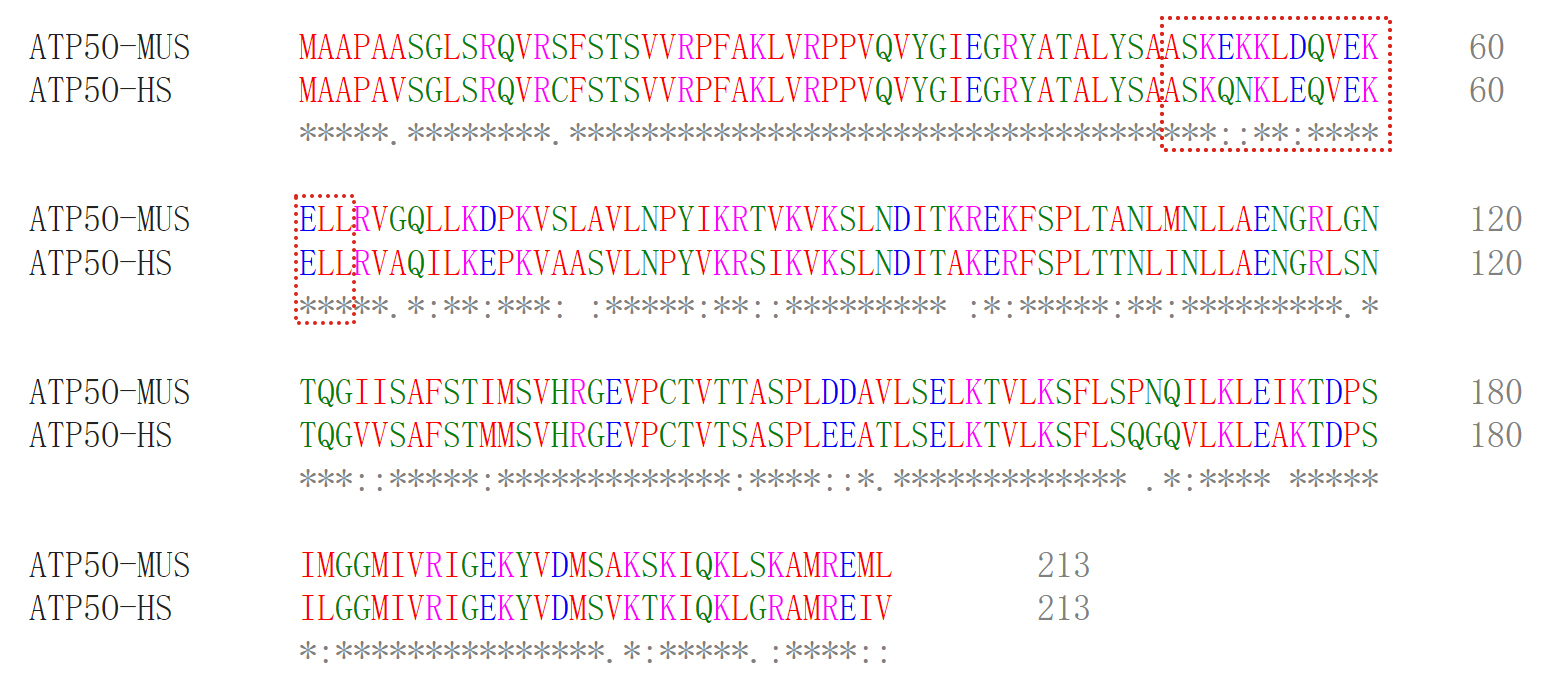

Supplement: Supplementary Materials — Supplementary figure 1: pan-crotonylation level was the most downregulated in the hypothalamus of CS mice. Supplementary figure 2: pan-crotonylation level was the most downregulated in the liver of CS mice Supplementary figure 3: pan-crotonylation level was the most downregulated in the ovary of CS mice. Supplementary figure 4: there were no significant differences for various pan-PTMs in the kidney. Supplementary figure 5: there were no significant differences for pan-crotonylation in various tissues. Supplementary figure 6: plasma of clinical females with high HAM-A scores has downregulated crotonylation. Supplementary figure 7: pan-crotonylation was still the most downregulated in preabsorbed plasma of clinical females with high HAM-A scores. Supplementary figure 8: there were no significant differences for the lipid metabolism-related enzymes in the kidneys. Supplementary figure 9: TAC is pharmacologically active and the TAC crotonylation was reversible. Supplementary figure 10: TAC sequence is highly specific for ATP5O. Supplementary figure 11: TAC is highly specific for ATP5O-K51cr. Supplementary figure 12: THP sequence is highly specific for HDAC2. Supplementary figure 13: protein sequence alignment between mouse and human ATP5O. Supplementary dataset 1, Supplementary dataset 2, Supplementary dataset 3, Supplementary dataset 4, Supplementary dataset 5, and Supplementary dataset 6. Supplementary table 1: radom mating table Supplementary table 2. Primer for qPCR Supplementary table 3. Plasmid construction primers for mouse HDAC2 and ATP5O Supplementary table 4. Mutagenesis primers of HDAC2 and ATP5O Supplementary table 5. In-vitro ubiquitination reaction components. [file 9834963.f1.zip › Supplementary figure 13.jpg]

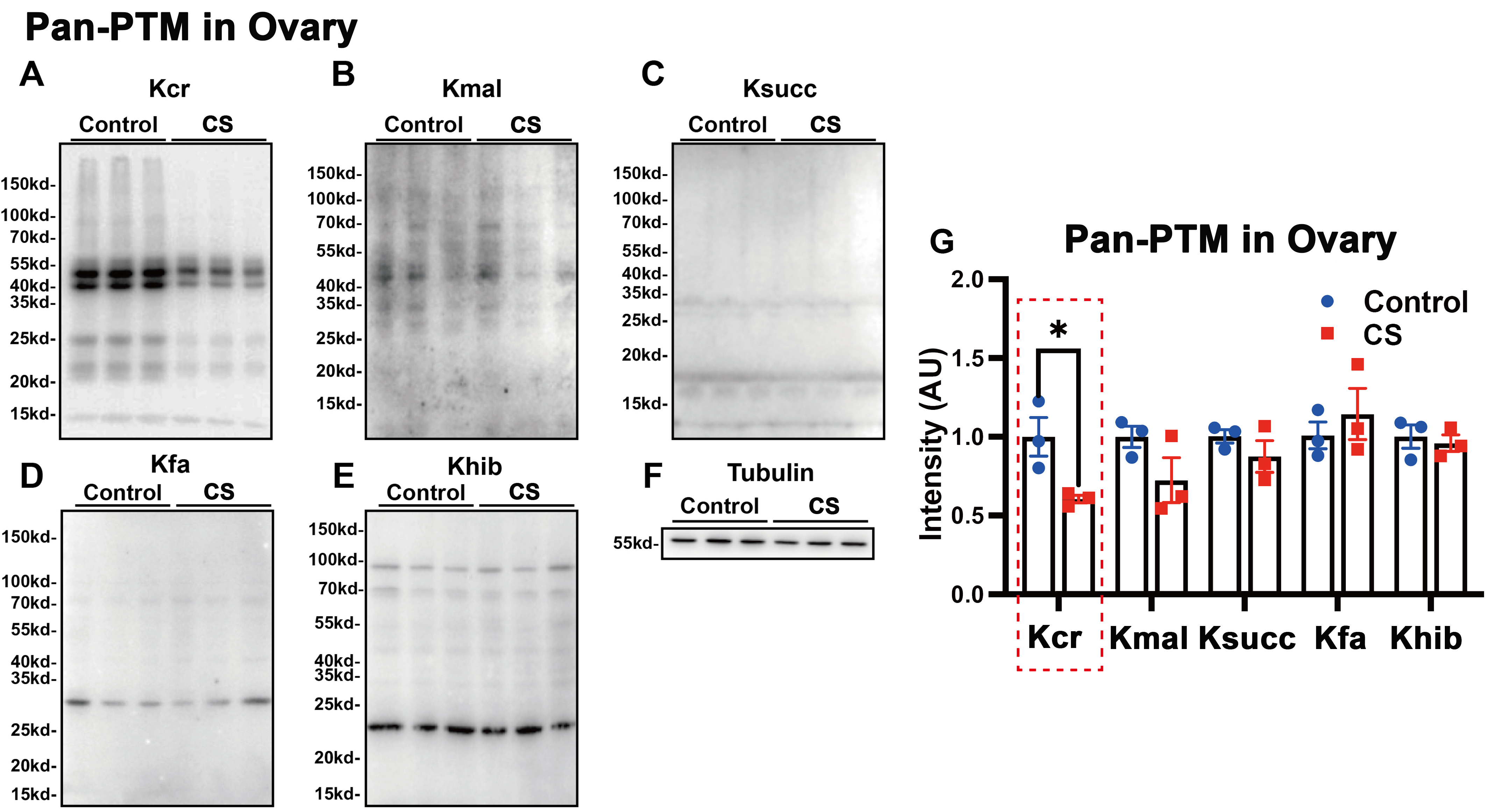

Supplement: Supplementary Materials — Supplementary figure 1: pan-crotonylation level was the most downregulated in the hypothalamus of CS mice. Supplementary figure 2: pan-crotonylation level was the most downregulated in the liver of CS mice Supplementary figure 3: pan-crotonylation level was the most downregulated in the ovary of CS mice. Supplementary figure 4: there were no significant differences for various pan-PTMs in the kidney. Supplementary figure 5: there were no significant differences for pan-crotonylation in various tissues. Supplementary figure 6: plasma of clinical females with high HAM-A scores has downregulated crotonylation. Supplementary figure 7: pan-crotonylation was still the most downregulated in preabsorbed plasma of clinical females with high HAM-A scores. Supplementary figure 8: there were no significant differences for the lipid metabolism-related enzymes in the kidneys. Supplementary figure 9: TAC is pharmacologically active and the TAC crotonylation was reversible. Supplementary figure 10: TAC sequence is highly specific for ATP5O. Supplementary figure 11: TAC is highly specific for ATP5O-K51cr. Supplementary figure 12: THP sequence is highly specific for HDAC2. Supplementary figure 13: protein sequence alignment between mouse and human ATP5O. Supplementary dataset 1, Supplementary dataset 2, Supplementary dataset 3, Supplementary dataset 4, Supplementary dataset 5, and Supplementary dataset 6. Supplementary table 1: radom mating table Supplementary table 2. Primer for qPCR Supplementary table 3. Plasmid construction primers for mouse HDAC2 and ATP5O Supplementary table 4. Mutagenesis primers of HDAC2 and ATP5O Supplementary table 5. In-vitro ubiquitination reaction components. [file 9834963.f1.zip › Supplementary figure 3.jpg]

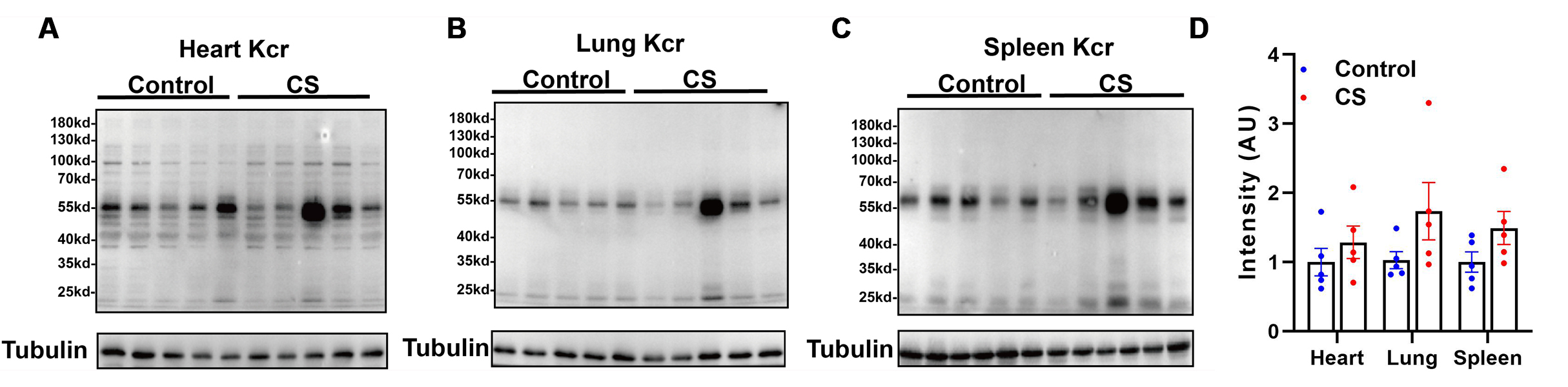

Supplement: Supplementary Materials — Supplementary figure 1: pan-crotonylation level was the most downregulated in the hypothalamus of CS mice. Supplementary figure 2: pan-crotonylation level was the most downregulated in the liver of CS mice Supplementary figure 3: pan-crotonylation level was the most downregulated in the ovary of CS mice. Supplementary figure 4: there were no significant differences for various pan-PTMs in the kidney. Supplementary figure 5: there were no significant differences for pan-crotonylation in various tissues. Supplementary figure 6: plasma of clinical females with high HAM-A scores has downregulated crotonylation. Supplementary figure 7: pan-crotonylation was still the most downregulated in preabsorbed plasma of clinical females with high HAM-A scores. Supplementary figure 8: there were no significant differences for the lipid metabolism-related enzymes in the kidneys. Supplementary figure 9: TAC is pharmacologically active and the TAC crotonylation was reversible. Supplementary figure 10: TAC sequence is highly specific for ATP5O. Supplementary figure 11: TAC is highly specific for ATP5O-K51cr. Supplementary figure 12: THP sequence is highly specific for HDAC2. Supplementary figure 13: protein sequence alignment between mouse and human ATP5O. Supplementary dataset 1, Supplementary dataset 2, Supplementary dataset 3, Supplementary dataset 4, Supplementary dataset 5, and Supplementary dataset 6. Supplementary table 1: radom mating table Supplementary table 2. Primer for qPCR Supplementary table 3. Plasmid construction primers for mouse HDAC2 and ATP5O Supplementary table 4. Mutagenesis primers of HDAC2 and ATP5O Supplementary table 5. In-vitro ubiquitination reaction components. [file 9834963.f1.zip › Supplementary figure 5.jpg]

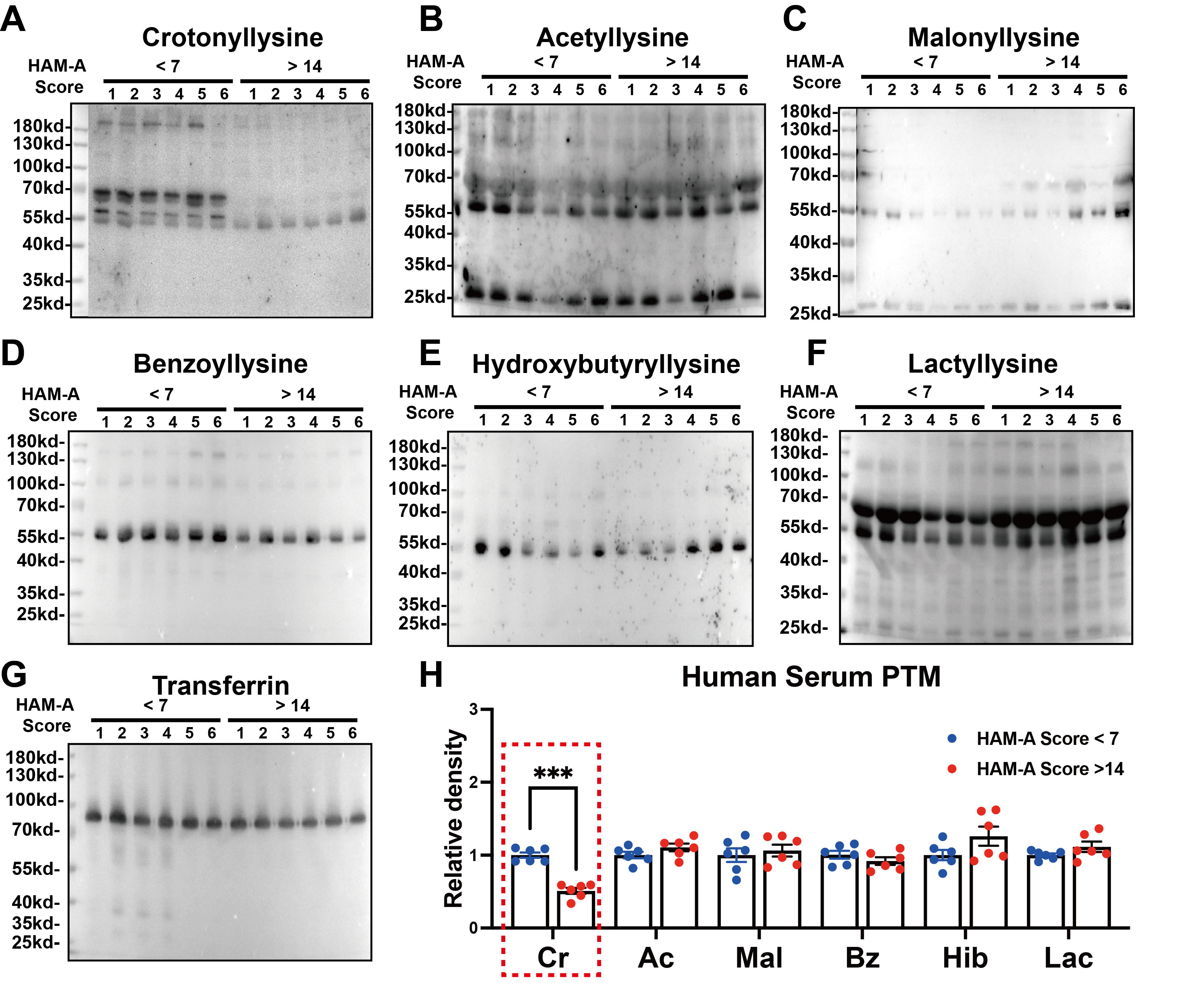

Supplement: Supplementary Materials — Supplementary figure 1: pan-crotonylation level was the most downregulated in the hypothalamus of CS mice. Supplementary figure 2: pan-crotonylation level was the most downregulated in the liver of CS mice Supplementary figure 3: pan-crotonylation level was the most downregulated in the ovary of CS mice. Supplementary figure 4: there were no significant differences for various pan-PTMs in the kidney. Supplementary figure 5: there were no significant differences for pan-crotonylation in various tissues. Supplementary figure 6: plasma of clinical females with high HAM-A scores has downregulated crotonylation. Supplementary figure 7: pan-crotonylation was still the most downregulated in preabsorbed plasma of clinical females with high HAM-A scores. Supplementary figure 8: there were no significant differences for the lipid metabolism-related enzymes in the kidneys. Supplementary figure 9: TAC is pharmacologically active and the TAC crotonylation was reversible. Supplementary figure 10: TAC sequence is highly specific for ATP5O. Supplementary figure 11: TAC is highly specific for ATP5O-K51cr. Supplementary figure 12: THP sequence is highly specific for HDAC2. Supplementary figure 13: protein sequence alignment between mouse and human ATP5O. Supplementary dataset 1, Supplementary dataset 2, Supplementary dataset 3, Supplementary dataset 4, Supplementary dataset 5, and Supplementary dataset 6. Supplementary table 1: radom mating table Supplementary table 2. Primer for qPCR Supplementary table 3. Plasmid construction primers for mouse HDAC2 and ATP5O Supplementary table 4. Mutagenesis primers of HDAC2 and ATP5O Supplementary table 5. In-vitro ubiquitination reaction components. [file 9834963.f1.zip › Supplementary figure 6.jpg]

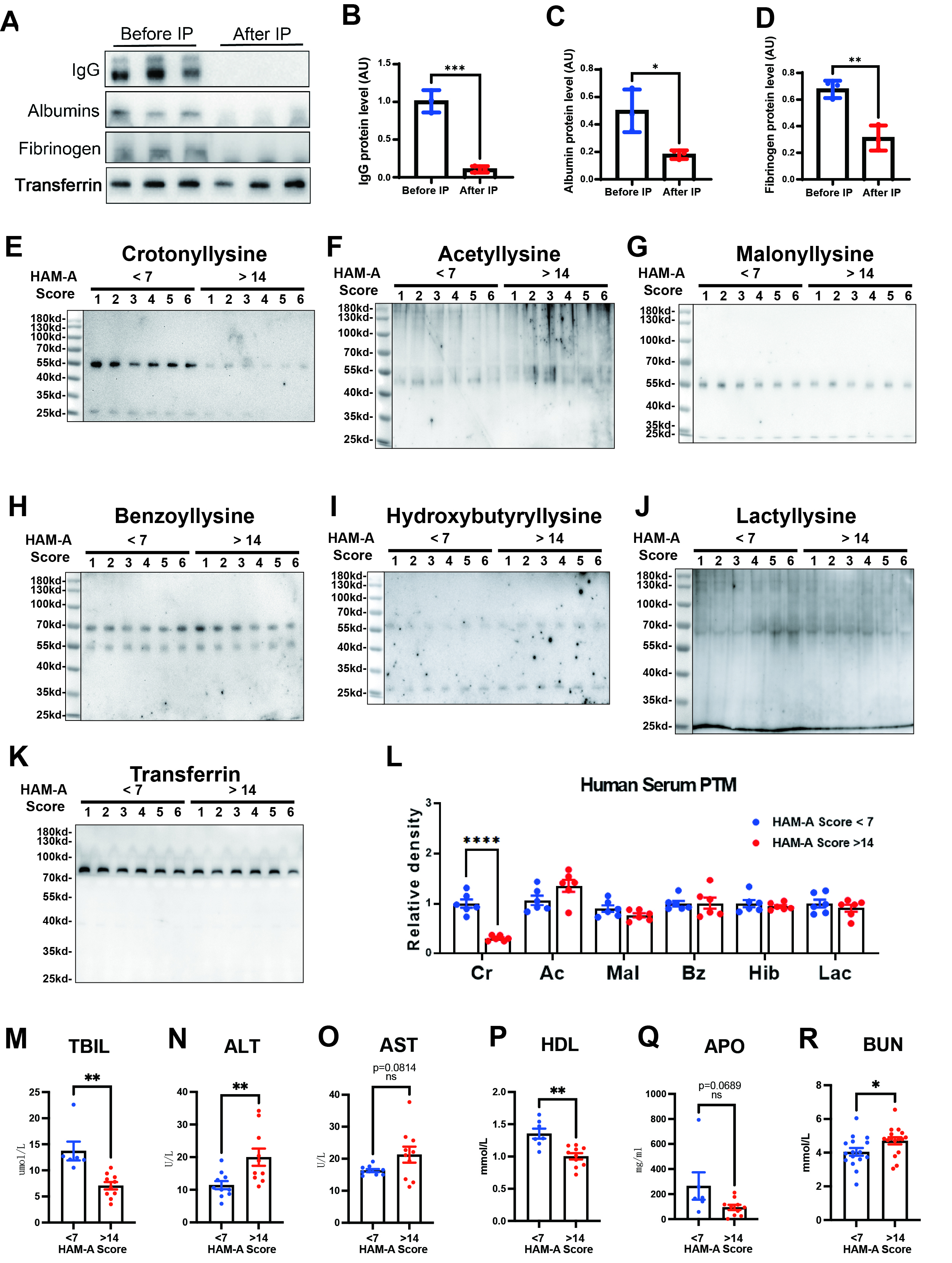

Supplement: Supplementary Materials — Supplementary figure 1: pan-crotonylation level was the most downregulated in the hypothalamus of CS mice. Supplementary figure 2: pan-crotonylation level was the most downregulated in the liver of CS mice Supplementary figure 3: pan-crotonylation level was the most downregulated in the ovary of CS mice. Supplementary figure 4: there were no significant differences for various pan-PTMs in the kidney. Supplementary figure 5: there were no significant differences for pan-crotonylation in various tissues. Supplementary figure 6: plasma of clinical females with high HAM-A scores has downregulated crotonylation. Supplementary figure 7: pan-crotonylation was still the most downregulated in preabsorbed plasma of clinical females with high HAM-A scores. Supplementary figure 8: there were no significant differences for the lipid metabolism-related enzymes in the kidneys. Supplementary figure 9: TAC is pharmacologically active and the TAC crotonylation was reversible. Supplementary figure 10: TAC sequence is highly specific for ATP5O. Supplementary figure 11: TAC is highly specific for ATP5O-K51cr. Supplementary figure 12: THP sequence is highly specific for HDAC2. Supplementary figure 13: protein sequence alignment between mouse and human ATP5O. Supplementary dataset 1, Supplementary dataset 2, Supplementary dataset 3, Supplementary dataset 4, Supplementary dataset 5, and Supplementary dataset 6. Supplementary table 1: radom mating table Supplementary table 2. Primer for qPCR Supplementary table 3. Plasmid construction primers for mouse HDAC2 and ATP5O Supplementary table 4. Mutagenesis primers of HDAC2 and ATP5O Supplementary table 5. In-vitro ubiquitination reaction components. [file 9834963.f1.zip › Supplementary Figure 7.jpg]

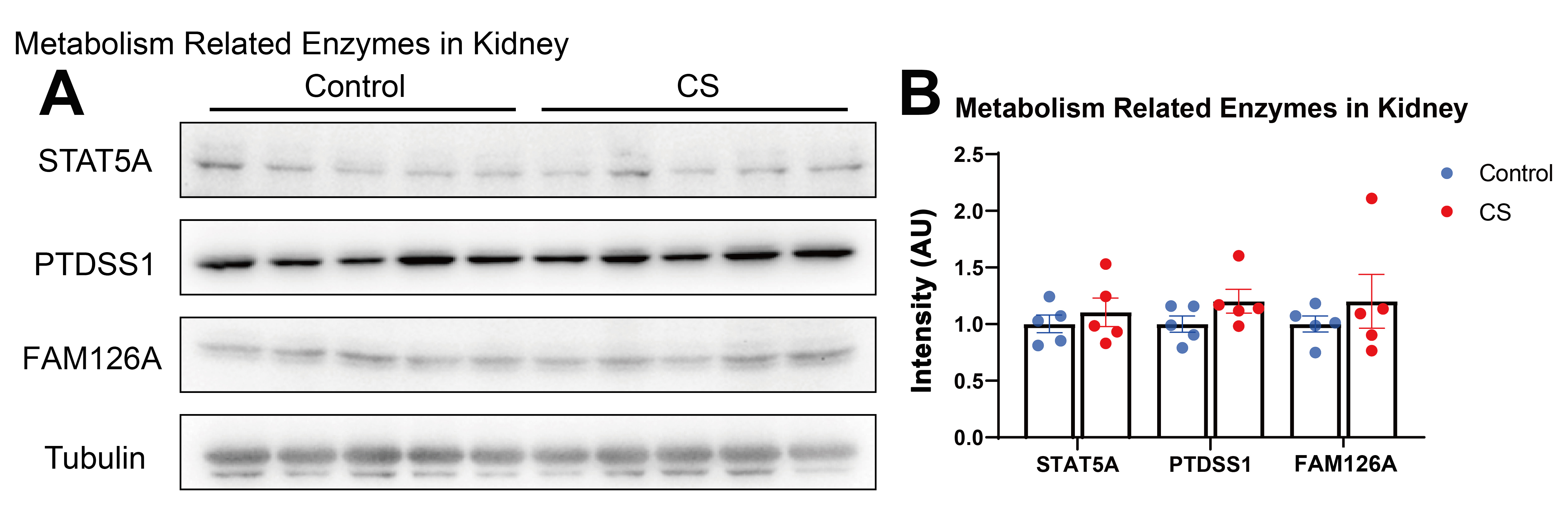

Supplement: Supplementary Materials — Supplementary figure 1: pan-crotonylation level was the most downregulated in the hypothalamus of CS mice. Supplementary figure 2: pan-crotonylation level was the most downregulated in the liver of CS mice Supplementary figure 3: pan-crotonylation level was the most downregulated in the ovary of CS mice. Supplementary figure 4: there were no significant differences for various pan-PTMs in the kidney. Supplementary figure 5: there were no significant differences for pan-crotonylation in various tissues. Supplementary figure 6: plasma of clinical females with high HAM-A scores has downregulated crotonylation. Supplementary figure 7: pan-crotonylation was still the most downregulated in preabsorbed plasma of clinical females with high HAM-A scores. Supplementary figure 8: there were no significant differences for the lipid metabolism-related enzymes in the kidneys. Supplementary figure 9: TAC is pharmacologically active and the TAC crotonylation was reversible. Supplementary figure 10: TAC sequence is highly specific for ATP5O. Supplementary figure 11: TAC is highly specific for ATP5O-K51cr. Supplementary figure 12: THP sequence is highly specific for HDAC2. Supplementary figure 13: protein sequence alignment between mouse and human ATP5O. Supplementary dataset 1, Supplementary dataset 2, Supplementary dataset 3, Supplementary dataset 4, Supplementary dataset 5, and Supplementary dataset 6. Supplementary table 1: radom mating table Supplementary table 2. Primer for qPCR Supplementary table 3. Plasmid construction primers for mouse HDAC2 and ATP5O Supplementary table 4. Mutagenesis primers of HDAC2 and ATP5O Supplementary table 5. In-vitro ubiquitination reaction components. [file 9834963.f1.zip › Supplementary figure 8.jpg]

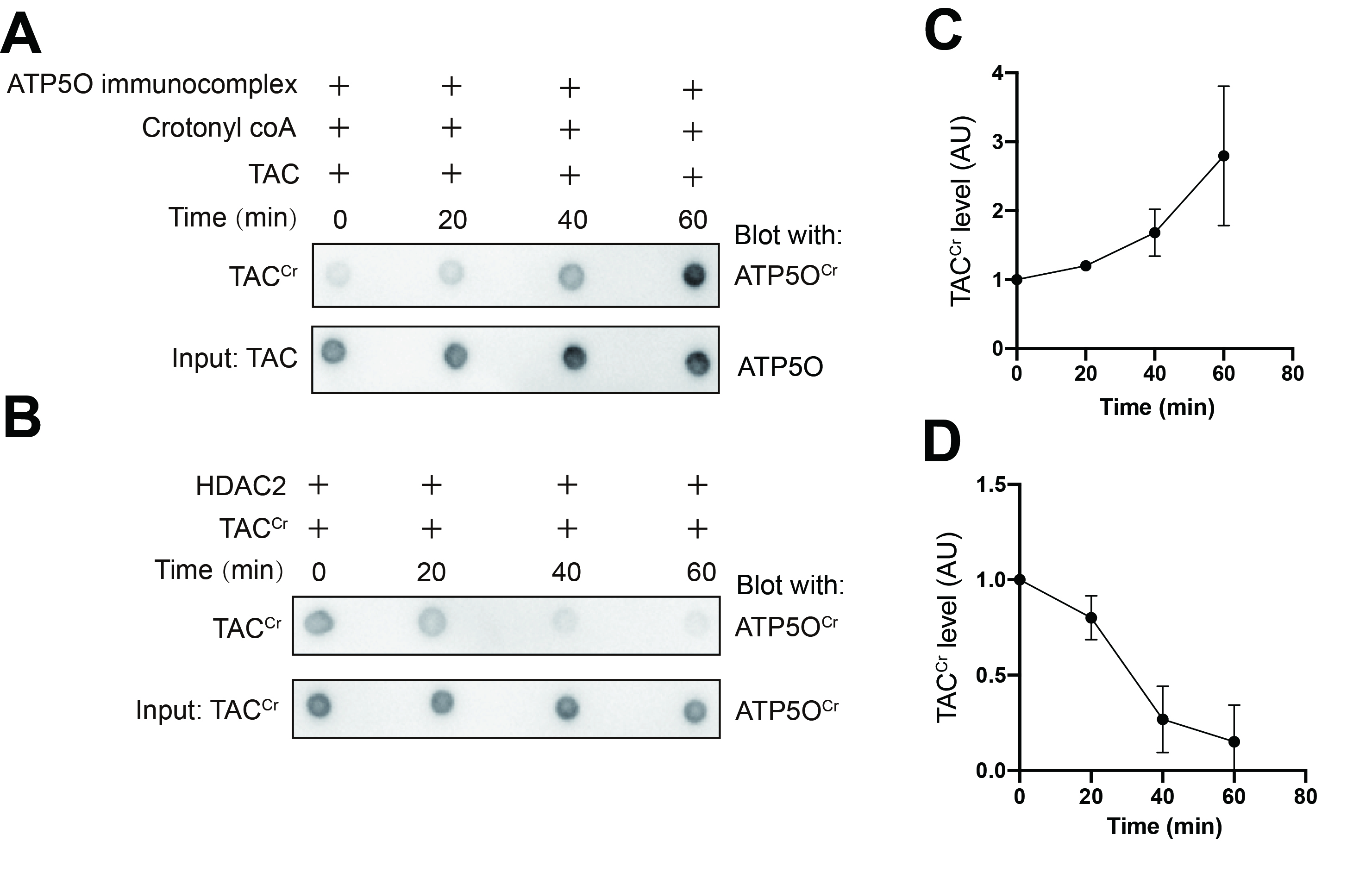

Supplement: Supplementary Materials — Supplementary figure 1: pan-crotonylation level was the most downregulated in the hypothalamus of CS mice. Supplementary figure 2: pan-crotonylation level was the most downregulated in the liver of CS mice Supplementary figure 3: pan-crotonylation level was the most downregulated in the ovary of CS mice. Supplementary figure 4: there were no significant differences for various pan-PTMs in the kidney. Supplementary figure 5: there were no significant differences for pan-crotonylation in various tissues. Supplementary figure 6: plasma of clinical females with high HAM-A scores has downregulated crotonylation. Supplementary figure 7: pan-crotonylation was still the most downregulated in preabsorbed plasma of clinical females with high HAM-A scores. Supplementary figure 8: there were no significant differences for the lipid metabolism-related enzymes in the kidneys. Supplementary figure 9: TAC is pharmacologically active and the TAC crotonylation was reversible. Supplementary figure 10: TAC sequence is highly specific for ATP5O. Supplementary figure 11: TAC is highly specific for ATP5O-K51cr. Supplementary figure 12: THP sequence is highly specific for HDAC2. Supplementary figure 13: protein sequence alignment between mouse and human ATP5O. Supplementary dataset 1, Supplementary dataset 2, Supplementary dataset 3, Supplementary dataset 4, Supplementary dataset 5, and Supplementary dataset 6. Supplementary table 1: radom mating table Supplementary table 2. Primer for qPCR Supplementary table 3. Plasmid construction primers for mouse HDAC2 and ATP5O Supplementary table 4. Mutagenesis primers of HDAC2 and ATP5O Supplementary table 5. In-vitro ubiquitination reaction components. [file 9834963.f1.zip › Supplementary figure 9.jpg]
