# Supplementary material for: ATP5O Hypo-crotonylation Caused by HDAC2 Hyper-Phosphorylation Is a Primary Detrimental Factor for Downregulated Phospholipid Metabolism under Chronic Stress
Source: Research (Wash D C). 2022 Nov 24;2022:9834963. doi: 10.34133/2022/9834963 (PMC11030818; doi:10.34133/2022/9834963)
Supplement: Supplementary Materials — Supplementary figure 1: pan-crotonylation level was the most downregulated in the hypothalamus of CS mice. Supplementary figure 2: pan-crotonylation level was the most downregulated in the liver of CS mice Supplementary figure 3: pan-crotonylation level was the most downregulated in the ovary of CS mice. Supplementary figure 4: there were no significant differences for various pan-PTMs in the kidney. Supplementary figure 5: there were no significant differences for pan-crotonylation in various tissues. Supplementary figure 6: plasma of clinical females with high HAM-A scores has downregulated crotonylation. Supplementary figure 7: pan-crotonylation was still the most downregulated in preabsorbed plasma of clinical females with high HAM-A scores. Supplementary figure 8: there were no significant differences for the lipid metabolism-related enzymes in the kidneys. Supplementary figure 9: TAC is pharmacologically active and the TAC crotonylation was reversible. Supplementary figure 10: TAC sequence is highly specific for ATP5O. Supplementary figure 11: TAC is highly specific for ATP5O-K51cr. Supplementary figure 12: THP sequence is highly specific for HDAC2. Supplementary figure 13: protein sequence alignment between mouse and human ATP5O. Supplementary dataset 1, Supplementary dataset 2, Supplementary dataset 3, Supplementary dataset 4, Supplementary dataset 5, and Supplementary dataset 6. Supplementary table 1: radom mating table Supplementary table 2. Primer for qPCR Supplementary table 3. Plasmid construction primers for mouse HDAC2 and ATP5O Supplementary table 4. Mutagenesis primers of HDAC2 and ATP5O Supplementary table 5. In-vitro ubiquitination reaction components. [file 9834963.f1.zip › Chen LJ et al-final revision-supp. info.-clean file.doc]

**ATP5O Hypo-crotonylation Caused by HDAC2 Hyper-phosphorylation is a Primary Detrimental Factor for Downregulated Phospholipid Metabolism under Chronic Stress**

Liang-Jian Chen^1, 4, 7^, Zhi-Yuan Tu^2, 1, 7^, Yang Wang^1, 7^, Yu-Hao He^1, 7^, Xin Wang^1^, Shu-Zhen Tao^2^, Yang-Yang Xu^2^, Cong-Rong Li^1^, Ruo-Lei Wang^1^, Zhi-Xia Yang^1^, Jing Sun^3, 6^, Xiang Ma^2, 1, 6^, and Dong Zhang^1, 5, 6^

^1^ State Key Lab of Reproductive Medicine, Nanjing Medical University, Nanjing, 211166, Jiangsu, China

^2^ Reproductive Medical Center, Jiangsu Province Hospital affiliated to Nanjing Medical University, Nanjing, 211166, Jiangsu, China

^3^ Department of Psychiatry, Nanjing Brain Hospital affiliated to Nanjing Medical University, Nanjing, 210029, Jiangsu, China

^4^ Department of Obstetrics and Gynecology, Reproductive Medicine Center, The First Affiliated Hospital of Anhui Medical University, Hefei, 230022, China.

^5^ Animal Core Facility, Nanjing Medical University, Nanjing, 211166, Jiangsu, P.R.China

^6^ To whom correspondence should be addressed to:

**Dong Zhang:** State Key Lab of Reproductive Medicine, Nanjing Medical University, 101 Longmian Ave., Nanjing, 211166, Jiangsu, China; dong.ray.zhang@njmu.edu.cn; Tel. +86-25-86869504; Fax. +86-25-86869504.

**Xiang Ma:** Reproductive Medicine Center, Jiangsu Provincial People's Hospital, First Affiliated to Nanjing Medical University, 16 Yongqing lane, Nanjing, 210000, Jiangsu, China; xiangma@njmu.edu.cn; Tel. +86-25-68302222; Fax. +86-25-68302222.

**Jing Sun:** Department of Psychiatry, Nanjing Brain Hospital affiliated to Nanjing Medical University, 264 Guangzhou Road, Nanjing, 210024, Jiangsu, China; jingsun2007@163.com; Tel. +86-25-82296218; Fax. +86-25-83719457.

**^7^** Equal contribution

**Running title**

ATP5O Hypo-crotonylation Disrupts Lipid Metabolism

**Supplementary MATERRIALS AND METHODS**

**Oocyte collection and in vitro culture**

Fully-grown GV oocytes were collected from control or CS female mice. Oocytes were released by puncturing follicles with a sterile syringe needle in a MEM+ medium (0.01 mM EDTA, 0.23 mM Na-pyruvate, 0.2 mM penicillin / streptomycin and 3 mg / ml BSA in MEM). After washing away the cumulus cells from the cumulus-oocyte complexes, every 50 oocytes were cultured in 100 µl mini-drops of MEM+ containing 20% fetal bovine serum (FBS) (Thermo Fisher) covered with mineral oil at 37.0 °C in an incubator with 5% O_2_, 5% CO_2_, and a humidified atmosphere.

**Immunofluorescence staining of oocytes**

50 oocytes per repeat were permeabilized with 0.5% Triton X-100 / PHEM (60 mM PIPES, 25 mM Hepes, pH 6.9, 10 mM EGTA, 8 mM MgSO_4_) for five minutes, and then were fixed in 3.7% FPA in PHEM for 20 minutes at room temperature. After being washed with PBS / 0.05% PVP (polyvinylpyrrolidone) three times at 10 minutes each, oocytes were blocked in blocking buffer (100 mM glycine and 1% BSA in PBS) for one hour at room temperature. Primary antibodies were then diluted in blocking buffer and oocytes were incubated in it overnight at 4.0 °C. The following steps were performed as described above.

**Ovarian hematoxylin-eosin staining and ovary follicle counting**

2-month-old control and CS female mice were used for follicle counting. To eliminate the effects of distinct estrous cycles among different mice, 10 IU / mice PMSG were intraperitoneally injected into mice to synchronize the estrous cycle. Ovaries were obtained after 48 hours, washed, and fixed in 10% buffered formalin or 4% PFA overnight, embedded in paraffin, continuously sectioned at 5 µm thickness, then stained with hematoxylin and eosin. The follicle stages were classified according to Pederson's standard. In brief, an oocyte surrounded by a single layer of flattened or cubical granulosa cell was defined as a primordial or primary follicle; an oocyte surrounded by more than one layer of cuboidal granulosa cells without visible antrum was defined as a secondary follicle; a follicle possessing a clearly antral space containing follicular fluid was defined as an antral follicle. For the follicle number, only follicles with visible nuclei were counted. And we counted every other two slices (since the same follicle will appear at adjacent different slices) and the final follicle number of a certain stage is a cumulative number of all counts of the corresponding stage. The number of follicles counted per ovary was used for statistical analysis.

**Co-immunoprecipitaion**

2.5 μg rabbit anti-HDAC2 antibody, rabbit anti-ATP5O antibody, rabbit anti-ATP5A1 antibody, or rabbit anti-ATP5B antibody was first coupled to 30 μl protein-A/G beads (Yeasen, Shanghai, China) for 4 hours at 4°C on a rotating wheel in 250 μl immunoprecipitate (IP) buffer (20 mM Tris-HCl, pH 8.0, 10 mM EDTA, 1 mM EGTA, 150 mM NaCl, 0.05% Triton X-100, 0.05% Nonidet P-40, 1 mM phenylmethylsulfonyl fluoride) with 1:100 protease inhibitor (Sigma) and 1:500 phosphatase inhibitor (Sigma). Meanwhile, 400 oocytes were lysed and ultrasonicated in 250 µl IP buffer and then pre-cleaned with 30 µl protein-A/G beads for 4 hours at 4 °C. After that, protein A/G-coupled Rabbit IgG or specific antibody were incubated overnight at 4 °C with pre-cleaned oocyte lysate supernatant. Finally, after being washed three times (10 minutes each with 250 µl IP buffer), the resulting beads with bound immunocomplexes were subjected to SDS-PAGE and western blot.

**Plasmid construction**

All to-be-expressed gene fragments were amplified by High-fidelity DNA polymerase (Vazyme) from cDNA reverse-transcribed by SSRT VI (Thermo Fisher), then the fragments are digested by high-fidelity restriction enzymes (NEB), purified, and inserted into pcDNA3.1 (+) plasmid by Quick ligase (NEB). All primer sequences for the constructs are in Supp. table 3. In all construct, HDAC2 or ATP5O (WT or mutant, all mutagenesis primers are in Supp. table 4) was fused to flag or strepII for rapid detection with flag or strepII antibody.

**Expression and purification of ATP5O and HDAC2 in SF9 cells.**

EGFP-strepII-tagged ATP5O-WT, ATP5O to A (K51A), ATP5O to Q (K51Q) and TagRFP-flag-tagged HDAC2-WT, HDAC2 to A (S424A), HDAC2 to D (S424D) proteins were cloned and expressed through a Bac-to-bac system (Thermo Fisher). In brief, corresponding sequences (Supplementary Table 4) were cloned into pFastBacHTA and then transformed into DH10Bac E.coli cells. The bacmid was isolated from DH10Bac E.coli (Vazyme) with QIAfilter Plasmid Purification kit (QIAGEN) and then transfected into Sf9 cells (Genetimes ExCell Technology, cat. no. ATCC CRL-3357) with Cellfectin II Transfection reagent (Thermo Fisher) in the static upper plane of an orbital shaker (Shanghai Zhichu Instrument) at 27 °C. The virus-containing supernatant was used to infect fresh Sf9 cells for 72 h and the procedure was repeated twice to amplify the baculovirus. Then, 20 μl of the third cycle supernatant was added to Sf9 cells in 250 ml SFM900-II medium (Thermo Fisher) with 5% FBS to express proteins in the lower bed of an orbital shaker at 27 °C and 200 rpm. Next, infected cells were resuspended in a lysis buffer (containing 50 mM Tris, 10% sucrose, 50 µM ATP, 1 mM phenylmethylsulfonyl fluoride (PMSF), 5 mM dithiothreitol (DTT), 1% NP40, 10 mM imidazole, 1×protease inhibitor and phosphatase inhibitor, pH 7.0 by HCl) and lysed with a high-pressure cell disrupter (Union Biotech), centrifuged and the resulting supernatant was incubated with 1 ml Ni-NTA Superflow resin (QIAGEN) at 4 °C for 1 h. The resin was then transferred into a 5-ml chromatography column (Biocomma), washed with four column volumes of wash buffer (40 mM imidazole in resuspension buffer without PMSF) and eluted with 500 mM imidazole in resuspension buffer without PMSF. The eluted protein was concentrated by a size-exclusion spin column and exchanged into BRB80 (80 mM HEPES, 1 mM MgCl2 and 1 mM EGTA, pH 6.8 by KOH) with 10% glycerol, 50 μM ATP and 5 mM DTT. The protein was aliquoted and kept at −80 °C for future use.

**Expression and purification of GST-ATP5O proteins in E.coli.**

For a GST-ATP5O, the recombined ATP5O sequence was cloned into pGEX6P1 and transformed into BL21-DE3 competent E.coli (Vazyme). Transformed E.coli was grown in 4 × 1000 ml LB+ medium in an orbital shaker (Shanghai Zhichu Instrument Co.) at 37 °C and 220 rpm. until OD600 reached 0.6~1.0. Next, 0.2 mM isopropyl-b-d-thiogalactoside (IPTG) was added into the medium to induce the recombined ATP5O expression overnight at 16 °C, 220 rpm. The next morning, E.coli was spun down, washed once with cold PBS, lysed and purified as described above.

**Pull-down assay**

Purified ATP5O-GST fusion protein was incubated with HDAC2-WT in GST lysis buffer (1 × PBS, 0.5 mM DTT, 1% Trixon-100, 0.1 mM PMSF, 1 mM ATP, protease inhibitor and phosphatase inhibitor cocktails (1:500, Yeasen), 1 M MgSO_4_, 1M EGTA) for 1.5 h at room temperature. After adding 100 μl of GST sepharose Resin (BBI, Sangon Biotech) and incubation for 1.5 h at room temperature, beads were washed three times with 45 ml of GST lysis buffer, after washing three times with GST lysis buffer, the bound proteins were subjected to SDS-PAGE and stained with Coomassie blue.

**Assay of mitochondrial transmembrane potential**

50 oocytes per repeat were incubated at 37 °C for 20 min with the fluorescent potentiometric indicator JC-1 (Cat#: 40706ES60, Yeasen, Shanghai, China) diluted 1:200, then washed twice with PBS and added to droplets (50 µl) of culture medium. Images of green fluorescence (JC-1 as monomers at low membrane potentials) and red fluorescence (JC-1 as aggregates at higher membrane potentials) were captured using confocal microscopy as above. Mitochondrial depolarization is indicated by a decrease in the red / green fluorescence intensity ratio.

**Detection of ROS generation**

The ROS Assay Kit (Cat#: S0033, Beyotime, Beijing, China) was used to detect ROS generation in oocytes. In brief, 50-100 oocytes per repeat were incubated with a dichlorofluorescein diacetate (DCFH-DA) probe for 20 min at 37 °C in the dark, washed, and mounted on slides for confocal imaging.

**Quantification of ROS, JC-1, immunofluorescence, western blot, and agarose gel images**

We always used original tif images for quantification of ROS, JC-1, immunofluorescence, western blot, or agarose gel images. The image is opened in image j (National Institutes of Health, Bethesda, MD, USA), For intensity measurement in ROS, JC-1, and immunofluorescence, signal intensity was first measured, then background intensity was also measured, the net intensity was obtained from signal intensity subtracted by background intensity. For gross intensity measurement in a western blot or agarose gel, the net intensity was obtained as above, then the band area was measured, finally, the integrated intensity was obtained from net intensity multiplied by band area.

**Supplementary Figure legends**

**Supplementary figure 1. Pan-Crotonylation level was the most downregulated in the hypothalamus of CS mice.**

A–G. Western blot in the CS (chronic stress) hypothalamus (Hth) showed that, among diverse pan-PTMs, pan-crotonylation level was the most significantly downregulated, whereas other major PTMs did not show significant decrease. Besides, there was a medium but significant upregulation for pan- acetylation. A and G, pan-crotonylation (Kcr); B and G, pan-acetylation (Kac); C and G, pan- benzoylation (Kben); D and G, pan-succinylation (Ksucc); E and G, pan- malonylation (Kmal). Tubulin was used as a loading control. * Indicates p ˂ 0.05.

**Supplementary figure 2. Pan-Crotonylation level was the most downregulated in the liver of CS mice**

A–G. Western blot in the CS liver showed that, among diverse pan-PTMs, pan-crotonylation level was the most significantly downregulated, whereas other major PTMs did not show significant decrease. A and G, pan-crotonylation (Kcr); B and G, pan-acetylation (Kac); C and G, pan- benzoylation (Kben); D and G, pan-succinylation (Ksucc); E and G, pan- malonylation (Kmal). Tubulin was used as a loading control. * Indicates p ˂ 0.05.

**Supplementary figure 3. Pan-Crotonylation level was the most downregulated in the ovary of CS mice**

A–G. Western blot in the CS ovaries showed that, among diverse pan-PTMs, pan-crotonylation level was the most significantly downregulated, whereas other major PTMs did not show significant differences. A and G, pan-crotonylation (Kcr); B and G, pan-malonylation (Kmal); C and G, pan-succinylation (Ksucc); D and G, pan-farnesylation (Kfa); E and G, pan-2-hydroxyisobutyrylation (Khib). Tubulin was used as a loading control. * Indicates p ˂ 0.05.

**Supplementary figure 4. There were no significant differences for various pan-PTMs in the kidney.**

A–G. Western blot showed that multiple pan-PTMs did not show significant differences in kidney between control and CS mice. A and G, pan-crotonylation (Kcr); B and G, pan-acetylation (Kac); C and G, pan- benzoylation (Kben); D and G, pan-succinylation (Ksucc); E and G, pan- malonylation (Kmal). Tubulin was used as a loading control.

**Supplementary figure 5. There were no significant differences for pan-crotonylation in various tissues.**

A-D. Western blot showed that there were no significant differences for pan- crotonylation (Kcr) in heart (A and D), lung (B and D), and spleen (C and D). Tubulin was used as a loading control.

**Supplementary figure 6. Plasma of clinical females with high HAM-A scores has downregulated crotonylation**

A-H. We apprised the HAM-A (Hamilton Rating Scale for Anxiety) scores and divided them into control (HAM-A score < 7), stress-prone (7 < HAM-A score < 14), or stress (HAM-A score > 14) groups. Plasma samples of the control and stress groups were collected to examine diverse PTMs. Results showed that pan-crotonylation was the most downregulated in CS female plasma, whereas the other PTMs were not changed. I-L. Blood biochemical indexes showed that the levels of TBIL, ALT, HDL, and BUN were all significantly different. Transferrin was used as a loading control.

**Supplementary figure 7. Pan-crotonylation was still the most downregulated in pre-absorbed plasma of clinical females with high HAM-A scores**

A-D. The highly rich proteins such as immunoglobulin (IgG), albumin, and fibrinogen within plasma were pre-depleted through immunoprecipitation, then their levels were detected by western blot. Quantification showed that IgG, albumin, and fibrinogen significantly decreased in pre-depleted plasma. E-L. Western blot and quantification showed that pan-crotonylation was the most downregulated in pre-depleted CS female plasma, whereas the other PTMs were not changed. M-R. Blood biochemical indexes showed that the levels of TBIL, ALT, HDL, and BUN were all significantly different between normal and CS female plasma. Transferrin was used as a loading control.

**Supplementary figure 8.** **There were no significant differences for the lipid metabolism-related enzymes in the kidneys.**

A and B. Western blot showed that no significant difference for the lipid metabolism-related enzymes including STAT5A, FAM126A and PTDSS1 in the kidneys of CS group. Tubulin was used as a loading control.

**Supplementary figure 9.** **TAC is pharmacologically active and the TAC crotonylation was reversible**

A and B. In vitro crotonylation, western blot, and quantification showed that TAC can be crontonylated at K51 time-dependently by an ATP5O immunocomplex. C and D. In vitro de-crotonylation, western blot, and quantification showed that crotonylated TAC (TAC^cr^) can be de-crontonylated at K51 time-dependently by HDAC2.

**Supplementary figure 10.** **TAC sequence is highly specific for ATP5O.**

This table includes top 10 proteins that share the highest similarity with TAC peptide based on blast in protein database. Alignment, coverage percentage, and similarity percentage are shown. The protein with the highest coverage and similarity, Ubinuclein, was testified in Fig. 4H and 4I.

**Supplementary figure 11.** **TAC is highly specific for ATP5O-K51cr**

A and B. Pan-crotonylation western blot and quantification showed that in a ATP5O-depleted cell lysate, TAC supplement didn’t change the pan-crotonylation level at all, supporting its specificity for ATP5O-K51^cr^.

**Supplementary figure 12.** **THP sequence is highly specific for HDAC2.**

This table includes top 10 proteins that share the highest similarity with THP peptide based on blast in protein database. Alignment, coverage percentage, and similarity percentage are shown. The protein with the highest coverage and similarity, HDAC1-S421^p^, was testified in Fig. 5G.

**Supplementary figure 13.** **Protein sequence alignment between mouse and human ATP5O**

Protein sequence alignment between mouse and human ATP5O showed that although they are quite similar overall, at the region corresponding to the antigen sequence of ATP5O-K51^cr^ antibody (red dot-line boxed), they are somewhat different from each other.

**SUPPLEMENTARY DATASET legends**

**Supplementary dataset 1**

This file corresponds to Fig. 1L–N. Label-free crotonylome was employed to identify all crotonylated sites & proteins from control or CS mouse ovaries, two repeats each. This file includes two sheets. " All Site_quant Normalized" sheet include all identified crotonylated sites, normalized by gross protein level; "Stress vs control > 1.3 or < 0.77" sheet includes all crotonylated sites that were 1.3-fold upregulated or 0.77-fold downregulated in stress group in contrast to control.

**Supplementary dataset 2**

This file corresponds to Fig. 3B–E. Full-spectrum metabolome was employed to identify all types of metabolites from control or CS mice (six repeats each). This file includes two sheets. "All data" sheet include all identified metabolites; " S vs C > 1.5 or < 0.67" sheet includes all metabolites that were 1.5-fold upregulated or 0.67-fold downregulated in CS group in contrast to control.

**Supplementary dataset 3**

This file corresponds to Fig. 4M-O. Full-spectrum metabolome was employed to identify all types of metabolites from control (11 repeats) or Control + TAC mice (12 repeats). This file includes two sheets. "All data" sheet include all identified metabolites; "TAC vs C >1.5 or < 0.667" sheet includes all lipid metabolites that were 1.5-fold upregulated or 0.667-fold downregulated in TAC group in contrast to control.

**Supplementary dataset 4**

This file corresponds to Fig. 5A–C. TMT-labeled quantitative phosphoproteome was employed to identify all phosphorylated sites & proteins from control or CS mouse ovaries, two repeats each. This file includes two sheets. " All Site_quant Normalized" sheet include all identified phosphorylated sites, normalized by gross protein level; "Stress vs control > 1.2 or < 0.833" sheet includes all phosphorylated sites that were 1.2-fold upregulated or 0.833-fold downregulated in stress group in contrast to control.

**Supplementary dataset 5**

This file corresponds to Fig. 6F–H. Lipid metabolome was employed to identify all lipid metabolites from control, CS, or CS+THP-treated mice (six repeats each). This file includes two sheets. "ALL_DATA" sheet includes all identified lipid metabolites; "S vs SH > 1.5 or < 0.67" sheet includes all lipid metabolites that were 1.5-fold upregulated or 0.67-fold downregulated in CS+THP group in contrast to CS group and meanwhile close to control group.

**Supplementary dataset 6**

This file corresponds to Fig. 7F-H. Lipid metabolome was employed to identify all lipid metabolites of human serum from control (C), stress-prone (SP), or stress (S) groups (ten repeats each). This file includes two sheets. "ALL_DATA" sheet includes all identified lipid metabolites; " HCS vs HC > 1.5 or < 0.67" sheet includes all lipid metabolites that were 1.5-fold upregulated or 0.67-fold downregulated in S (HCS) group in contrast to control.

**Supplementary tables**

**Supplementary table 1. Radom mating table**

| **Male mouse No.** | **Mating cage No.** | | | | | | | |
| --- | --- | --- | --- | --- | --- | --- | --- | --- |
|  | **2019/10/10** | **2019/11/10** | **2019/12/10** | **2020/1/10** | **2020/2/10** | **2020/3/10** | **2020/4/10** | **2020/5/10** |
| 1# | C | L | G | T | E | T | A | T |
| 2# | D | O | A | N | I | S | H | R |
| 3# | B | S | C | R | G | K | E | K |
| 4# | A | R | E | S | J | P | F | L |
| 5# | H | T | B | L | C | R | I | M |
| 6# | E | Q | I | K | F | N | C | S |
| 7# | I | N | H | P | H | M | G | Q |
| 8# | G | M | F | O | B | Q | D | P |
| 9# | F | P | J | M | D | L | J | O |
| 10# | J | K | D | Q | A | O | B | N |
| 11# | L | B | K | J | N | F | Q | A |
| 12# | K | A | M | E | P | B | O | H |
| 13# | P | J | P | I | S | I | L | E |
| 14# | R | D | R | H | T | D | M | F |
| 15# | M | F | T | D | M | A | K | I |
| 16# | O | E | S | B | Q | J | T | C |
| 17# | N | G | Q | G | O | G | N | G |
| 18# | S | C | O | F | K | E | R | D |
| 19# | T | I | L | A | R | H | P | J |
| 20# | Q | H | N | C | L | C | S | B |

Supplementary table 2. Primer for qPCR

| **Name** | **Sequence** |
| --- | --- |
| *RFRP-*qPCR-F | TGATGCCTCATTTTCACAGCA |
| *RFRP-*qPCR-R | CTGCGGGGCTTCTTTTCTC |
| *GnRH-qPCR-F* | AGCACTGGTCCTATGGGTTG |
| *GnRH-qPCR-R* | CTTCACTCGGGAGCCTAAGCC |
| *GPR147-qPCR-F* | CCGGTTGGCCTTTTGACAAT |
| *GPR147-qPCR-R* | CAGCTTCTCACGGAAAGGGT |
| *ATP5O-qPCR-F* | TCTCGACAGGTTCGGAGCTT |
| *ATP5O-qPCR-R* | AGAGTACAGGGCGGTTGCATA |
| *Actin-qPCR-F* | CCGTAAAGACCTCTATGCC |
| *Actin-qPCR-R* | CTCAGTAACAGTCCGCCTA |

This table includes all primer for qPCR.

**Supplementary table 3. Plasmid construction primers for mouse *HDAC2* and *ATP5O***

| **Name** | **Sequence** | **Length** |
| --- | --- | --- |
| Kpn1-Flag-HDAC2-F in pcDNA3.1(+) | CGGGGTACCGCCACCATGGACTATAAAGACGATGACGATAAAGCGTACAGTCAAGGAGGCG | 1464bp |
| BamH1-HDAC2-R in pcDNA3.1 (+) | CGCGGATCCAGGGTTGCTGAGTTGTTCTGACTTG |  |
| Kpn1-Strepii-ATP5O-F in pcDNA3.1(+) | CGGGGTACCGCCACCATGTGGAGTCATCCACAGTTCGAGAAGGCCGCGCCTGCAGCGTCCGGAC | 639bp |
| BamH1-ATP5O-R in pcDNA3.1 (+) | CGCGGATCCGAGCATCTCCCGCATGGCCTTGC |  |
| BamH1-EGFP-F in pcDNA3.1 (+) | CGCGGATCCGTGAGCAAGGGCGAGGAG | 717bp |
| Not1-EGFP-R in pcDNA3.1 (+) | AAGGAAAAAAGCGGCCGCTAACTTGTACAGCTCGTCCATGC |  |

This table includes all primers for plasmid construction of mouse *HDAC2 and ATP5O*

**Supplementary table 4. Mutagenesis primers of *HDAC2* and *ATP5O***

| **Name** | **Sequence** |
| --- | --- |
| *HDAC2*(S to A)-F | ATTCTCAGATGCTGAGGATGAAGGAGAAGGAGGTCGAAGAAAT |
| *HDAC2*(S to A)-R | CTTCATCCTCAGCATCTGAGAATTCTTCATCACAAGCTATCCG |
| *HDAC2*(S to D)-F | ATTCTCAGATGATGAGGATGAAGGAGAAGGAGGTCGAAGAAAT |
| *HDAC2*(S to D)-R | CTTCATCCTCATCATCTGAGAATTCTTCATCACAAGCTATCCG |
| *ATP5O*(K to A)-F | TGCTGCATCTGCGGAGAAGAAGCTGGACCAGGTGGAGAAGGAG |
| *ATP5O*(K to A)-R | GCTTCTTCTCCGCAGATGCAGCAGAGTACAGGGCGGTTGCATA |
| *ATP5O*(K to Q)-F | TGCTGCATCTCAAGAGAAGAAGCTGGACCAGGTGGAGAAGGAG |
| *ATP5O*(K to Q)-R | GCTTCTTCTCTTGAGATGCAGCAGAGTACAGGGCGGTTGCATA |

This table includes all primers for HDAC2 A or D mutant and ATP5O A or Q mutant.

**Supplementary table 5**. In-vitro ubiquitination reaction components

| **Components** | **Stock conc. (μM)** | **Final conc. (μM)** | **Volume (μl)** |
| --- | --- | --- | --- |
| 10 x reaction buffer | 10 x | 1 x | 1.5 |
| UBE1 (E1) | 5 | 0.2 | 0.6 |
| UbcH5a/UBE2D1 (E2) | 25 | 1 | 0.6 |
| Cul1/Rbx1 | 23 | 0.25 | 0.16 |
| Skp1/2 | 15 | 0.25 | 0.25 |
| Ubiquitin | 30 (μg/μl) | 30 (μg/μl) | 0.25 |
| ATP5O-E3 ubiquitin ligase immunecomplex | NA | NA | 1 |
| ATP | 75 | 1 | 0.2 |
| MgSO_4_ | 50 | 1 | 0.3 |
| ddH_2_0 | NA | NA | to 15 µl |

NA: not applicable
